# Multiple pore lining residues modulate water permeability of GlpF

**DOI:** 10.1002/pro.4431

**Published:** 2022-09-21

**Authors:** Kristyna Pluhackova, Valentin Schittny, Paul‐Christian Bürkner, Christine Siligan, Andreas Horner

**Affiliations:** ^1^ Stuttgart Center for Simulation Science, Cluster of Excellence EXC 2075 University of Stuttgart Stuttgart Germany; ^2^ Department of Biosystems Science and Engineering Eidgenössische Technische Hochschule (ETH) Zurich Basel Switzerland; ^3^ Institute of Biophysics Johannes Kepler University Linz Austria

**Keywords:** aquaporin, determinants of water permeability, gating, GlpF, pore lining residues, protein engineering, water permeability modulation

## Abstract

The water permeability of aquaporins (AQPs) varies by more than an order of magnitude even though the pore structure, geometry, as well as the channel lining residues are highly conserved. However, channel gating by pH, divalent ions or phosphorylation was only shown for a minority of AQPs. Structural and in silico indications of water flux modulation by flexible side chains of channel lining residues have not been experimentally confirmed yet. Hence, the aquaporin “open state” is still considered to be a continuously open pore with water molecules permeating in a single‐file fashion. Using protein mutations outside the selectivity filter in the aqua(glycerol)facilitator GlpF of *Escherichia coli* we, to the best of our knowledge, for the first time, modulate the position of the highly conserved Arg in the selectivity filter. This in turn enhances or reduces the unitary water permeability of GlpF as shown in silico by molecular dynamics (MD) simulations and in vitro with purified and reconstituted GlpF. This finding suggests that AQP water permeability can indeed be regulated by lipid bilayer asymmetry and the transmembrane potential. Strikingly, our long‐term MD simulations reveal that not only the conserved Arg in the selectivity filter, but the position and dynamics of multiple other pore lining residues modulate water passage through GlpF. This finding is expected to trigger a wealth of future investigations on permeability and regulation of AQPs among others with the aim to tune water permeability for biotechnological applications.

## 1 INTRODUCTION

Depending on age, the human body consists of up to 75% water. Body water homeostasis between lipid compart‐ments is regulated through a complex interplay of osmotically active substances, including ions and neutral solutes. The rate of water flow across such barriers is mediated and regulated by specialized proteins, called aquaporins (AQPs), optimized for osmotically induced passive water transport. Since their first discovery in 1992 by Agre and coworkers,^1^, ^2^ 13 different AQPs were discovered in mammals.^3^ AQPs are expressed in a wide variety of tissues throughout the human body, including the retina, inner ear, brain, spinal cord, blood vessels, heart, kidney, salivary glands, gastrointestinal tract, liver, pancreas, lungs, fat tissue, skin, and the reproductive system, where they are involved in a wide range of physiological functions.^4^ These functions include water/salt homeostasis, exocrine fluid secretion and epidermal hydration. Due to their important tasks throughout the body, AQPs are involved in various human diseases, including glaucoma, cancer, epilepsy, and obesity.^5^, ^6^ Mutations in their primary sequence cause genetic diseases like nephrogenic diabetes insipidus, congenital cataracts, and keratoderma. Besides their substantial role in mammals, AQPs fulfill pivotal functions in plants, where they are also involved in the regulation of cellular water homeostasis.^7^ This includes a key role in transpiration sensitivity to soil drying as well as to high atmospheric vapor pressure deficit.^8^ Furthermore, AQPs are also expressed in all other kingdoms of life (archea, eubacteria, and fungi). Interestingly, despite their enormous importance, very little was known about the molecular determinants of water flow through such single‐file transmembrane protein channels until recent methodological advancements enabled quantitative unitary permeability measurements.^9^, ^10^, ^11^

The narrow AQP pores combine enormous permeability, conducting water in a single‐file manner close to the diffusion limit of water in the bulk, with perfect selectivity.^11^, ^12^ Even protons are rejected by the concerted action of the two constriction sites^13^, ^14^: (a) the selectivity filter with its positively charged arginine and aromatic residues (ar/R) and (b) the dual asparagine–proline–alanine motive (NPA), aiding the rotation of water molecules upon reorientation along the counteracting dipole moment of the half helices.^14^, ^15^, ^16^ Interestingly, the NPA motives alone are sufficient for exclusion of alkali cations.^13^, ^14^ The major determinants of single‐file water transport across biological channels were suggested to be the number of hydrogen bonds that water molecules may form with channel lining residues^11^ and the presence of closed conformational states of the respective channel due to channel gating as well as possibly the geometry of the vestibules and positive charges at the channel's mouth.^9^, ^10^ The latter is probably connected with the energy penalty of water permeation from dehydration of water molecules entering the narrow pores.^10^, ^17^ A subset of AQPs, termed aquaglyceroporins (AQGP), possess broader and more hydrophobic pores.^18^ These enable the conduction of glycerol, urea, and other small organic molecules like glycine,^19^ and the neutral hydroxides As(OH)_3_ and Sb(OH)_3_, inorganic equivalents of polyols.^20^, ^21^ Nevertheless, even AQGP efficiently exclude proton permeation^22^ as exemplified by GlpF the prototypical AQGP from *Escherichia coli*. Polyol permeability through GlpF depends on the length (number of C‐atoms) and the stereochemistry of the sugar alcohol with glycerol exhibiting the highest mobility within the channel.^18^, ^19^ Glycerol conduction is accompanied by an inherent competition between water and glycerol for hydrogen bonds with channel lining residues with water being indispensable for glycerol conduction.^23^ The transport through GlpF was discovered to be mostly influenced by the ar/R selectivity filter,^18^, ^24^ while in sole water transporters, the largest energy barrier for water translation is localized at the two NPA motives.^25^ Water permeation through GlpF is highly debated as three experimental groups reported that the osmotic permeability of AQPZ, an orthodox water channel of *E. coli*, exceeds that of GlpF,^22^, ^26^, ^27^ whereas three theoretical groups observed the exact opposite.^28^, ^29^, ^30^, ^31^ Most recent experimental results including several methodological refinements^11^ reported osmotic permeability of GlpF exceeding the in silico estimations for AQPZ by a factor of 2,^30^ and that of GlpF by a factor of 12.^28^, ^30^ The high experimental water permeability of monomeric GlpF was confirmed by short molecular dynamics (MD) simulations under high osmotic pressure,^32^ with questionable physiological relevance as high pressure may distort the GlpF structure and thus disrupt the single‐file conformation of the water molecules inside.^28^ It is important to note here, that at much lower osmotic pressures, the water permeability through, for example, *Bt*AQP1 was experimentally shown to be independent of the applied osmotic pressure.^33^

Several eukaryotic AQPs have been proposed to be regulated by pH,^34^, ^35^ phosphorylation,^36^ divalent cation binding,^37^ or membrane‐mediated mechanical stress.^38^ Open or closed states can be caused by large‐scale rearrangements of cytoplasmic loops or the displacement of an aquaporin specific key‐residue.^39^, ^40^, ^41^, ^42^, ^43^, ^44^ Our recent MD simulations of *Bt*AQP1, *Hs*AQP4, *Ec*AQPZ, and *Ec*GlpF revealed three potential gating sides of pore lining residues moving into the pore lumen. These are the conserved arginine in the ar/R selectivity filter (R^h2.2^ using our recently introduced general numbering scheme^45^ visualized in Figure S1), the conserved histidine at the cytoplasmic pore end (H^h1.‐3^) and methionines M202/M212 in *Ec*GlpF/*Hs*AQP4 directly preceding the second NPA motif (M^h2.‐2^).^45^ Similar blocking motion for the conserved R^h2.2^ in the ar/R constriction region was reported before for *Ec*AQPZ^46^, ^47^ and *Bt*AQP1.^48^ Moreover, MD simulations have suggested that AQPs may be voltage‐gated by this arginine.^49^, ^50^, ^51^ By application of unphysiologically large voltages of 1.5 V the R^h2.2^ was found to be stabilized in an upstate by a positive potential, resulting in rapid water flux, whereas a negative potential induced a downstate reducing the channel permeability to water.^50^ Experimental evidence for AQP gating by pore lining residues is scarce. Alternative R^h2.2^ positions were found in structural studies of *Hs*AQP1^52^ and *Ec*AQPZ,^53^, ^54^ indicating the flexibility of this side chain and suggesting that different R^h2.2^ states, including the upstate and the downstate, may indeed be populated under physiological conditions. Alternation between two R^h2.2^ conformations is envisioned to disrupt continuous flow of water, thus regulating the open probability of the water pore.^54^ In contrast, NMR spectroscopy experiments revealed no indication of R189^h2.2^ gating in reconstituted *Ec*AQPZ^55^ and the *Rn*AQP1‐R195V^h2.2^ mutant did not show increased water permeability.^13^, ^14^ However, experiments on *Hs*AQP4 show that an H201E^5.5^ mutant, potentially forming a salt‐bridge with R216^h2.2^, reduces water flux and the R216A^h2.2^ mutant increases water permeability.^56^ But one has to keep in mind that mutations in the selectivity filter may lead to loss of pore selectivity and to the disruption of the pore structure by repositioning of the protein backbone and neighboring side chains. Interestingly, in the AQGP *Pf*AQP a glutamic acid (E125^4.‐20^) in the vicinity of the R^h2.2^ residue was suggested to stabilize the arginine in its open state.^57^ Such stabilization by a negatively charged amino acid is further corroborated by a mutagenesis of T120E^4.‐18^ in lens *Oa*AQP0 which suggests with 93% confidence that the permeability of the mutant is higher than that of the wild‐type (wt) protein.^58^ Our recent structural comparisons of 20 nonredundant high‐resolution AQP structures revealed a hydrogen bonding network of the R^h2.2^ involving up to seven pore lining residues.^45^ Thereby, the structurally found number of potential H‐bonds and H‐bonding partners of R^h2.2^ coincided with the occurrence of various arginine orientations in MD simulations of *Bt*AQP1, *Hs*AQP4, *Ec*AQPZ, and *Ec*GlpF. Hence, this hydrogen bonding network seems to have the potential to predict water permeability modulation within AQPs by the conserved R^h2.2^.

Here, in order to shed more light on the potential role of the conserved R^h2.2^ in modulating water permeability of AQPs and AQGPs, we conducted multi‐microsecond‐long MD simulations and in vitro water permeability measurements on the AQGP GlpF from *E. coli*. Thereby, *Ec*GlpF wt as well as mutants V29E^1.9^ and V29K^1.9^ were embedded in native like *E. coli* membranes both in silico^59^ and in vitro. The mutations located outside the single‐file region of the channel, at the periplasmic pore entrance, are designed to stabilize the conserved R^h2.2^ in an open or closed configuration and accordingly modifying water permeability through the channel.

## 2 RESULTS

**FIGURE 1:**
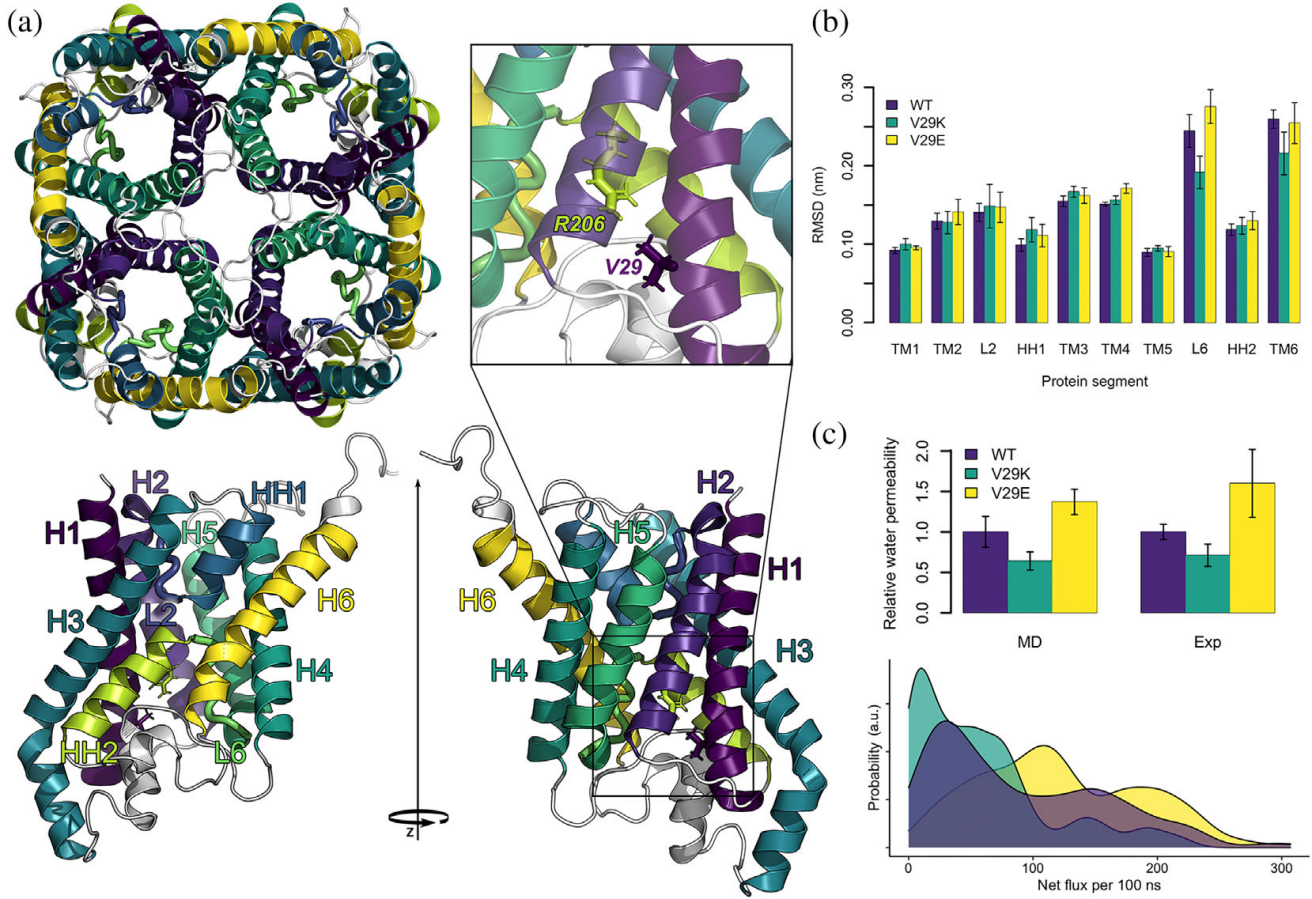
Structure, structural stability, and water permeability of the GlpF proteins. (a) Top left subfigure shows the tetrameric assembly of wild type (wt) GlpF seen from the cytoplasmic side. The bottom row visualizes monomeric GlpF from two side views, with the cytoplasmic side at the top and periplasmic side on the bottom. The six transmembrane helices H1–H6, the two half‐helices HH1 and HH2 and the two intracellular loops L2 and L6 are differently colored and labeled. The top right subfigure shows a close‐up view of R206^h2.2^ positioned at the HH2 and the mutation side V29^1.9^, located at the H1. (b) Average root mean square deviations of the individual protein segments estimated as the average deviation from 1 to 3 μs of the Cα atoms of each secondary structure element after a fit of the Cα atoms of the transmembrane bundle of the monomeric GlpF to the crystal structure 1FX8. The error bars give *SEM* of eight protomers. (c) Top plot shows relative water permeabilities of the mutants to the wt protein from molecular dynamics (MD) simulations (left) and experiments (right). In case of MD simulations, net water flux and in case of experiments osmotic water permeabilities were used for estimation of relative water permeabilities. The error bars in MD data give *SEM* of eight individual proteins. The error bars in the experiment give the error of the fit to three independent reconstitution series of five different protein to lipid ratios, each. Each reconstitution series was performed with newly purified protein. Bottom plot shows distributions of net flux of water molecules per 100 ns through the wt and mutant proteins during 1–3 μs of MD simulations.

Our previous MD simulations have revealed flexibility in the spontaneous positioning of the conserved R206^h2.2^ in the GlpF pore modulating water flow through the pore.^45^ In order to understand the impact of the different R206^h2.2^ positions on the water permeability of the protein, we have set here to study the water permeability of the wt GlpF and two mutants designed to influence the position of R206^h2.2^ by a joint experimental/in silico approach. By positioning of a negatively charged glutamic acid or positively charged lysine to the proximity of R206^h2.2^, that is, replacing V29^1.9^, (Figure 1a) we expected to manipulate the R206^h2.2^ position in the selectivity filter in a similar way like an external electrostatic potential does.^50^ Driven by the focused electric field in the narrow channel, the lysine should repulse the arginine and therefore force it more into the pore further restricting water passage through the channel and the glutamic acid should attract the arginine, thus widening the space for the single‐file water path and enhancing water passage.

Our multi‐microsecond all‐atom MD simulations of wt GlpF and of V29E and V29K mutants in *E. coli* PLE reveal that in the beginning of the production run simulations the mutants show lower number of transported water molecules as compared to wt GlpF, which can be accounted to a fast adaptation of the pore lining residues to the mutation during the equilibration simulations. In case of wt GlpF and V29K mutant, the number of transported water molecules decreased drastically during the course of the simulation for about 1 μs (Figure S2). Such decrease in water permeability, reported before to a smaller extend,^12^ hints to adaptation of the pore structure after removal of glycerol and could be a biological control mechanism of membrane water permeability. To illustrate the need of long MD simulations for GlpF, we observed on average 89.2 ± 5.5 water permeation events per wt GlpF protomer and per 100 ns between 1 and 3 μs, whereas in the first 500 ns, this value was almost three times larger (256.7 ± 6.0). The latter is in perfect agreement with literature data of 200 water molecules passing monomeric GlpF each 100 ns under similar conditions.^29^ In contrast, by large external pressure (200 till 400 MPa), which results in disruption of the water single‐file conformation in the pore, Zhu et al. observed 1,000–2,600 water passage events through each GlpF in their short simulations of 0.5 ns.^28^ Similarly, Wambo et al.^32^ applied an osmotic gradient of 0.63 M to the membrane resulting in 1095 permeation events per 100 ns. Overall, the trend of the average water permeability in between 1 and 3 μs has shown that V29E leads to 37% increase in water permeability compared to the wt protein while the introduction of lysine to the pore reduces the water permeability by 36% (Figure 1c).

To see if we can reproduce these results in vitro, we overexpressed wt GlpF as well as mutants V29E and V29K in *E. coli*, purified them using Ni^2+^ chromatography in the presence of octylglucoside and reconstituted them in *E. coli* polar lipid extract (PLE) vesicles.^11^, ^60^, ^61^ Next, we subjected the protein containing large unilamelar vesicles to a hyperosmotic gradient in a stopped‐flow device at 4°C and recorded the scattered light intensity of volume decrease over the time course of several seconds and fitted the data with our analytical solution^11^ to the differential equation relating *P*_f_ and water efflux from lipid vesicles under these conditions (Figure S3). To be able to report relative permeabilities of wt GlpF, V29E, and V29K, we estimated the reconstitution efficiency by counting the number of protein‐containing vesicles before and after detergent micellelation with fluorescence correlation spectroscopy (Figure S4). Our experiments clearly show that we are truly able to modulate water flux according to our design intention, which suggests that we are indeed able to provide the first clear experimental evidence for water permeability modulation of AQPs via the conserved Arg^h2.2^ in the ar/R selectivity filter. In detail, we have experimentally found an increase in single channel water permeability through V29E as compared to the wt GlpF by 60% and a decrease of the V29K mutant by 29%, which is perfectly in line with our in silico findings (Figure 1c).

Even though, the trend of the relative average water permeability in our MD simulations matches perfectly that of our experiments, the broad distributions of the number of water molecules passing individual pores of wt and mutant proteins (Figure 1c, bottom) hint to a complex regulation of water permeability. In order to reveal the molecular reasons for the modulated water permeability through GlpF and the impact of the V29^1.9^ mutations, we have analyzed the MD simulations in more detail. At first, we have estimated the structural stability of the proteins. Next, we have indicated pore lining residues which are able to rotate or flip into or out of the channel. Finally, we have fitted Bayesian generalized multilevel models enabling us to estimate the effects of the indicated pore lining residues on water permeability of the channel, while controlling all other pore lining residues.

The structural stability of the proteins embedded in our native‐like model of the *E. coli* PLE membrane^59^ was acquired by measuring the root mean square deviations (RMSD) of the Cα atoms of the transmembrane helical core of the tetrameric proteins. The values were similar for all proteins, reaching at most 0.2 nm (Figure S5), hinting to a stable protein fold. Next, we have estimated the average RMSD of the individual helices, as well as the two intrapore loops, connecting the half helices with the transmembrane helices (Figure 1b). Except for the loop L6 and the membrane exposed TM6, which does not carry any pore‐facing residues, the average RMSDs of the Cα atoms within a given secondary structure element were in the range between 0.1 and 0.17 nm pointing to a very similar structure compared to the crystal structure. The slightly increased RMSD of TM6 results from partial unfolding of the ends of the helix upon its adaptation for optimal interactions with the lipid headgroups. L6 (composed of residues L197^5.11^, T198^5.12^, G199^h2.‐5^, F200^h2.‐4^ [selectivity filter], A201^h2.‐3^, M202^h2.‐2^, and N203^h2.‐1^ [NPA motif]) also shows slightly higher structural flexibility, mainly in wt GlpF and the V29E mutant. As we will show later on, four out of those seven residues (i.e., F200^h2.‐4^, A201^h2.‐3^, M202^h2.‐2^, and N203^h2.‐1^) are responsible for the modulation of water permeability of the pore, albeit with different frequencies and modulation strength.

**FIGURE 2:**
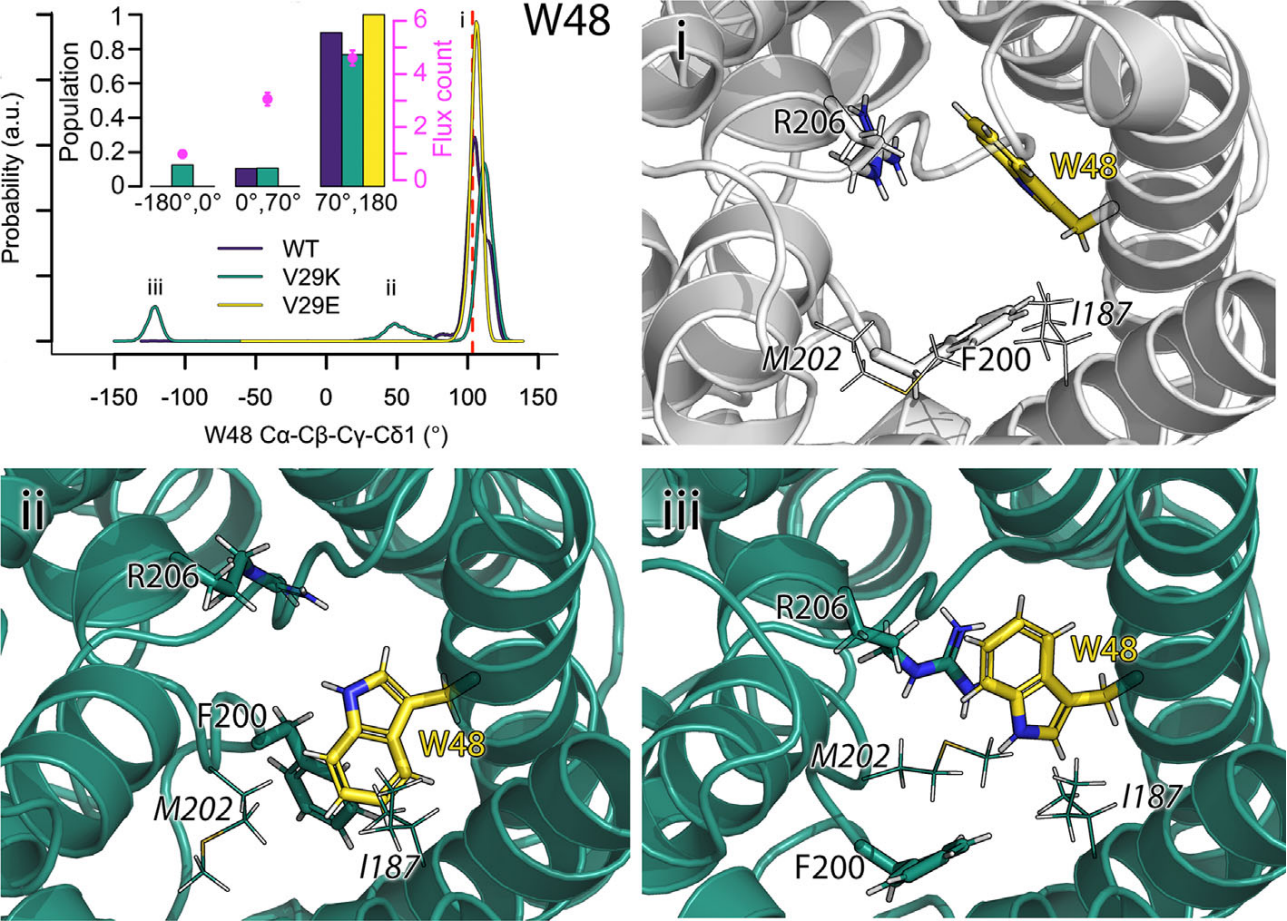
W48^2.‐3^ flipping into the pore lumen. The conformational flexibility of W48^2.‐3^ is described by the dihedral angle Cα‐Cβ‐Cγ‐Cδ1. The distributions for 1–3 μs of wild type (wt) GlpF (purple), V29K (green), and V29E (yellow) are shown in the top left panel. The populations of the states and predicted number of transported water molecules (flux count) over 5 ns in each state (i.e., −180° till 0°, 0° till 70°, and 70° till 180°) by our Bayesian model are depicted in the inset as bars and magenta points with error bars visualizing the 95% CI, respectively. The value of the dihedral angle in the crystal structure, visualized as gray cartoon in subfigure (i), is shown by a red dashed line in the distribution. (ii) Visualization of the W48^2.‐3^ dihedral angle of 50° in V29K shown as dark green cartoon. (iii) Visualization of the W48^2.‐3^ dihedral angle of −120° in V29K. The residues in the selectivity filter (W48^2.‐3^, F200^h2.‐4^, and R206^h2.2^) are shown as sticks, additional vicinal pore wall forming residues (I187^5.1^ and M202^h2.‐2^) are shown as lines. W48^2.‐3^ is highlighted in yellow.

Starting with the first member of the selectivity filter, W48^2.‐3^, we analyzed its flipping into the pore by estimating the dihedral angle Cα‐Cβ‐Cγ‐Cδ1 (Figure 2). In the crystal structure, the indole ring of W48^2.‐3^ adheres stably to the pore wall. This orientation, which is occupied for most of the simulation time in all simulation types, is characterized by a dihedral angle Cα‐Cβ‐Cγ‐Cδ1 of 103° and large water permeability (4.6 ± 0.3 water molecules per 5 ns). If the dihedral angle reduces to less than 70° but more than 0° W48^2.‐3^ partially obstructs the pore as indicated by a decreased water permeability to 3.0 ± 0.2 water molecules per 5 ns (panel ii of Figure 2). If the dihedral angle reaches about −120° W48^2.‐3^ almost completely blocks the pore as demonstrated by a water permeability of 1.0 ± 0.1 water molecules per 5 ns. While the restriction of the pore radius with decreased water permeability (i.e., W48^2.‐3^ dihedral angle between 0° and 70°) was detected for both, wt GlpF and the V29K mutant, for about 10% of the analyzed simulation time, the efficient blockage was found only in the V29K mutant (12.5% of the simulation time). Thereby both, temporary and rather persistent flipping events were observed (Figure S6). Similar behavior of W48^2.‐3^ was also witnessed in the 1‐μs‐long simulations of GlpF in a POPE membrane performed recently by Moss et al.^62^ In AQP2, F48^2.‐3^ located in the analogous position, was also seen capable of flipping into the pore and restricting water permeability.^63^ The fact that Moss et al^62^ observed flipping of W48^2.‐3^ into the pore of wt GlpF in a POPE membrane, while here, in a PE:PG:CL membrane W48^2.‐3^ flipped significantly into the pore only in case of the V29K mutant, hint to a possible charge induced modulation of GlpF permeability, for example, by membrane lipids. In this respect, it is interesting to mention that anionic lipids have been experimentally observed to modulate the activity of GlpF.^64^

**FIGURE 3:**
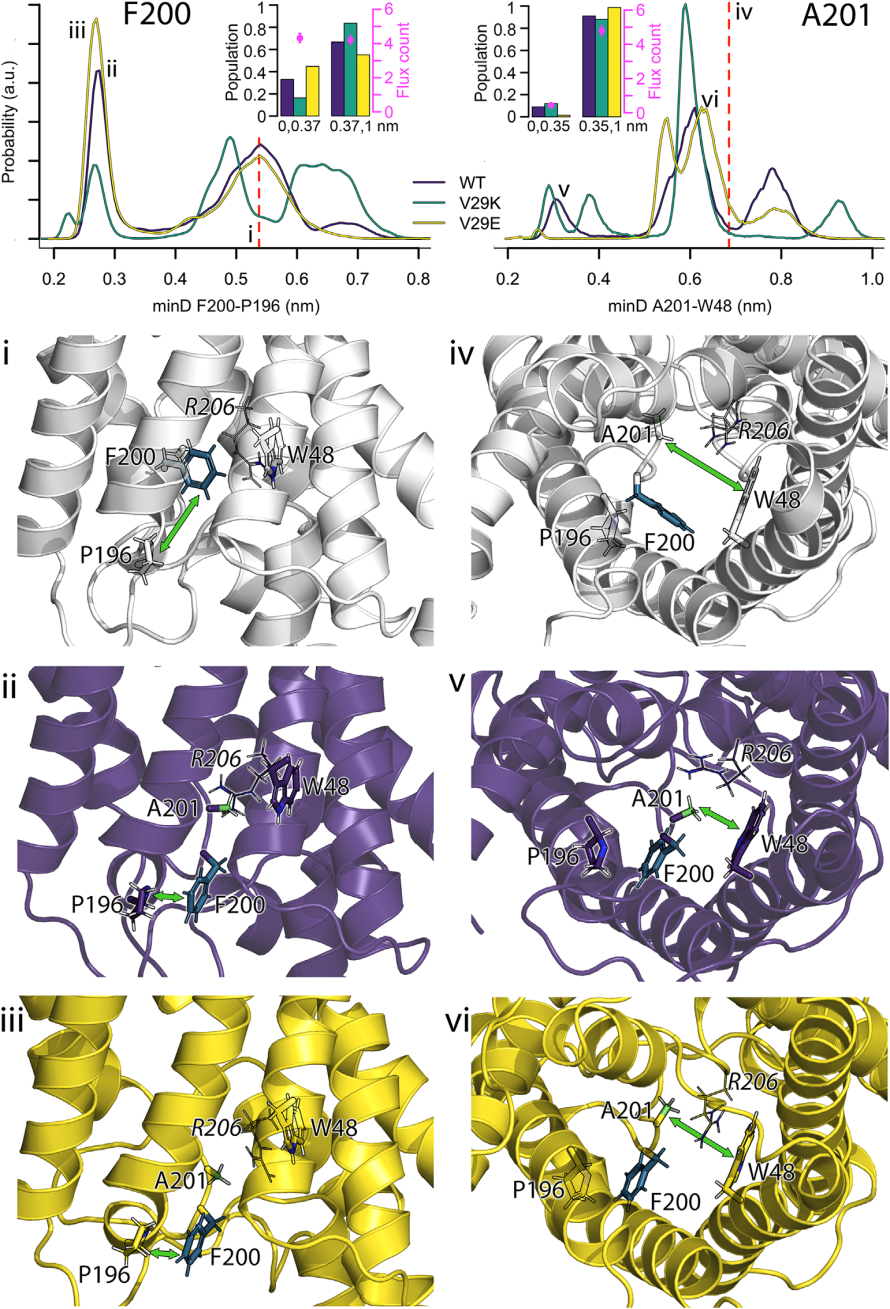
Conformation flexibility of F200^h2.‐4^ (left column) and A201^h2.‐3^ (right column) described by the minimal distance between the side chains of F200^h2.‐4^ to P196^5.10^ and A201^h2.‐3^ to W48^2.‐3^, respectively. Top row shows distributions of the minimal distances for wt GlpF (purple), V29K (green), and V29E (yellow) with the population of the individual states (i.e., distances shorter or longer than 0.37 and 0.35 nm, for F200^h2.‐4^‐P196^5.10^ and A201^h2.‐3^‐W48^2.‐3^, respectively) and the water permeability given as an average water flux count over 5 ns shown in the insets. The values of the minimal distances in the crystal structure are shown as red dashed lines in the distributions and visualized in subfigures (i) side view and (iv) cytoplasmic view. (ii) Side view on F200^h2.‐4^ (shown as dark green sticks) that flipped to P196^5.10^ (purple sticks) in a wt GlpF simulation (purple cartoon). At the same time, A201^h2.‐3^ (light green sticks) moved close to W48^2.‐3^ (purple sticks) which is best seen from the cytoplasmic view shown in subfigure (v). Subfigure (iii) shows a similar small distance between F200^h2.‐4^ and P196^5.10^ in a V29E simulations (yellow cartoon), but with A201^h2.‐3^ far away from W48^2.‐3^ (visualized in subfigure [vi] from the cytoplasmic view). The distances of interest are highlighted by green arrows.

The second member of the selectivity filter, F200^h2.‐4^, is able to flip away from the pore, thus potentially increasing the pore radius and enhancing water permeability. We have characterized this movement by measuring the minimum distance between the F200^h2.‐4^ and P196^5.10^ residues (Figure 3), that is, if F200^h2.‐4^ flips out of the pore the distance to P196^5.10^ is reduced. If the minimal distance amounts to less than 0.37 nm, the two residues are in close proximity. The flipping can be of a rather persistent nature (more than 1 μs), temporary (lasting tens to hundreds of nanoseconds) or floppy (the distance is rapidly switching between a short and a long one) as visualized in Figure S7. F200^h2.‐4^ moves most often to P196^5.10^ in the V29E mutant (44.7% of the simulation time), for 33.2% of simulation time in wt GlpF, and least often in case of the V29K mutant (16.3% of the simulation time). Such a movement resembles pore opening in AQP7 by F74^2.‐3,^ ^62^ (in position of W48^2.‐3^ in GlpF), where the flipping has been suggested to have a selectivity function by blocking the passage of small solutes but enabling glycerol transport by large‐scale displacement of F74^2.‐3^. Therefore, we were intrigued by the small difference in water permeability of the two states, that is, F200^h2.‐4^ close to P196^5.10^ is passed by 4.3 ± 0.3 and F200^h2.‐4^ far away from P196^5.10^ by 4.2 ± 0.3 water molecules each 5 ns. Visual investigations of our simulations have revealed that flipping of F200^h2.‐4^ to P196^5.10^ does not necessarily cause pore broadening, because other residues can move into the pore instead. One of these residues is A201^h2.‐3^, a direct neighbor of F200^h2.‐4^. In fact, this small residue is able to efficiently block the pore, if its side chain gets into a close proximity of W48^2.‐3^ (minimal distance between the side chains smaller than 0.35 nm, Figure 3, Figure S8, resulting in an average water permeability of 0.4 ± 0.0 water molecules per 5 ns, compared to 4.8 ± 0.3 water molecules passing if the distance is larger than 0.35 nm). While such a short, pore‐blocking distance between A201^h2.‐3^ and W48^2.‐3^ was rarely present in V29E (1.4%), it was well resolved in wt (9.1%) and even slightly more often occurring in V29K (12.0%). Interestingly, 2D maps of 100 ns intervals of average minimal distances between F200^h2.‐4^ and P196^5.10^ versus minimal distances between A201^h2.‐3^ and W48^2.‐3^ colored according to the average flux in the same time interval (Figures S9–S11) have revealed that F200^h2.‐4^ flipping to P196^5.10^ does not result in higher but in lower permeability of V29K mutant, while V29E exhibits often increased permeability at small F200^h2.‐4^‐P196^5.10^ distance, and in case of the wt protein, there is a mixture of low and intermediate permeabilities. This result hints to the fact that similar movement of the same residues does not have to result in the same impact on water permeability through an aqua(glycero)porin, as other residues may get involved.

**FIGURE 4:**
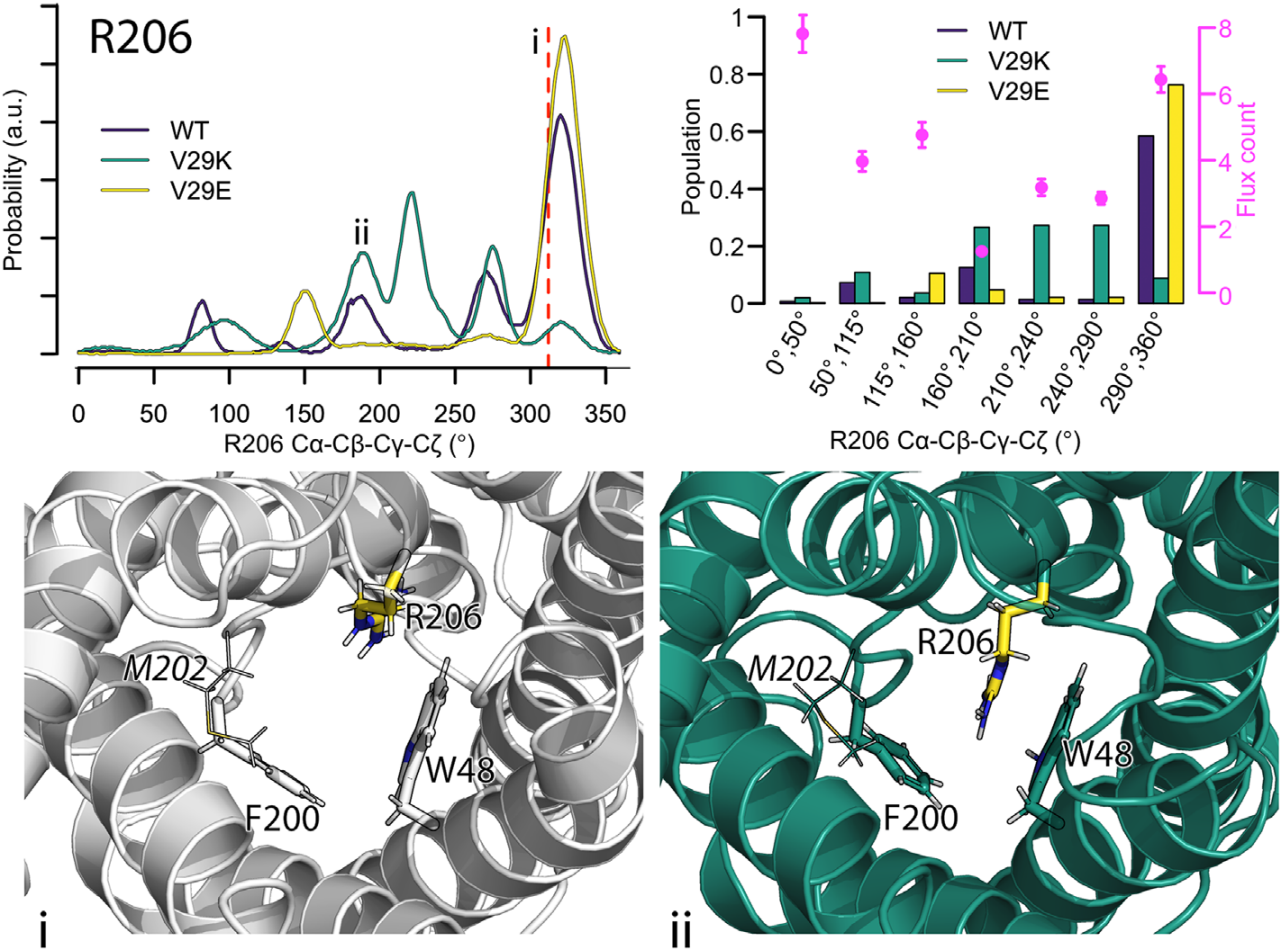
Positioning of R206^h2.2^ in the pore lumen. The orientational flexibility of R206^h2.2^ is followed by estimating its dihedral angle Cα‐Cβ‐Cγ‐Cζ. Top left figure shows the distributions of the R206^h2.2^ dihedral angle for wt GlpF and the mutants. The value found in the crystal structure (311°, visualized in subfigure (i)) is highlighted by a red dashed line. R206^h2.2^ occupying a dihedral angle of 200° in V29K (green cartoon) is shown in subfigure (ii). In both subfigures, R206^h2.2^ is highlighted in yellow, the other members of the selectivity filter (F200^h2.‐4^ and W48^2.‐3^) are shown as sticks and M202^h2.‐2^ is shown as lines. Top right figure shows populations of the different states of the R206^h2.2^ dihedral angle Cα‐Cβ‐Cγ‐Cζ and their water permeability per 5 ns estimated by our Bayesian model (magenta points with error bars visualizing the 95% CI).

The third member of the selectivity filter, R206^h2.2^, exhibits complex dynamics populating different states of the Cα‐Cβ‐Cγ‐Cζ dihedral angle with varying probability in the studied GlpF variants (Figure 4, Figures S12 and S13). Thereby, structures comprising angles between 290° and 360° (similar to the crystal structure with an angle of 311°) show water permeabilities of 6.4 ± 0.4 water molecules per 5 ns. This conformation is stabilized in the V29E mutant (76.3%) but strongly disfavored in the V29K mutation (8.8%) compared to 58.5% of the simulation time wt GlpF spends in this position. However, the pore is also (partially) open with R206^h2.2^ in other positions. In detail, positions between 0° and 160° show 7.8 ± 0.6 (0° ‐ 50°), 4.0 ± 0.3 (50° ‐ 115°) and 4.8 ± 0.4 (115° ‐ 160°) water permeation events per 5 ns while R206^h2.2^ found between 210° and 290° result in intermediate water permeabilities of 3.2 ± 0.3 (210°–240°) and 2.9 ± 0.2 (240°–290°) waters per 5 ns. In fact, the water permeability is drastically reduced to 1.3 ± 0.1 only for R206^h2.2^ dihedral angle in between 160° and 210°. This position and the two partially closed positions between 210° and 290° are most strongly populated in the V29K mutant (each with about 27%), while the V29E mutant strongly disfavors each of these conformations (with 5, 2, and 2% of the simulation time). wt GlpF also shows low preference for angles between 210° and 290°; however, it is more often found in the closed state of 160°–210° (12.6%) compared to the V29E mutant.

**FIGURE 5:**
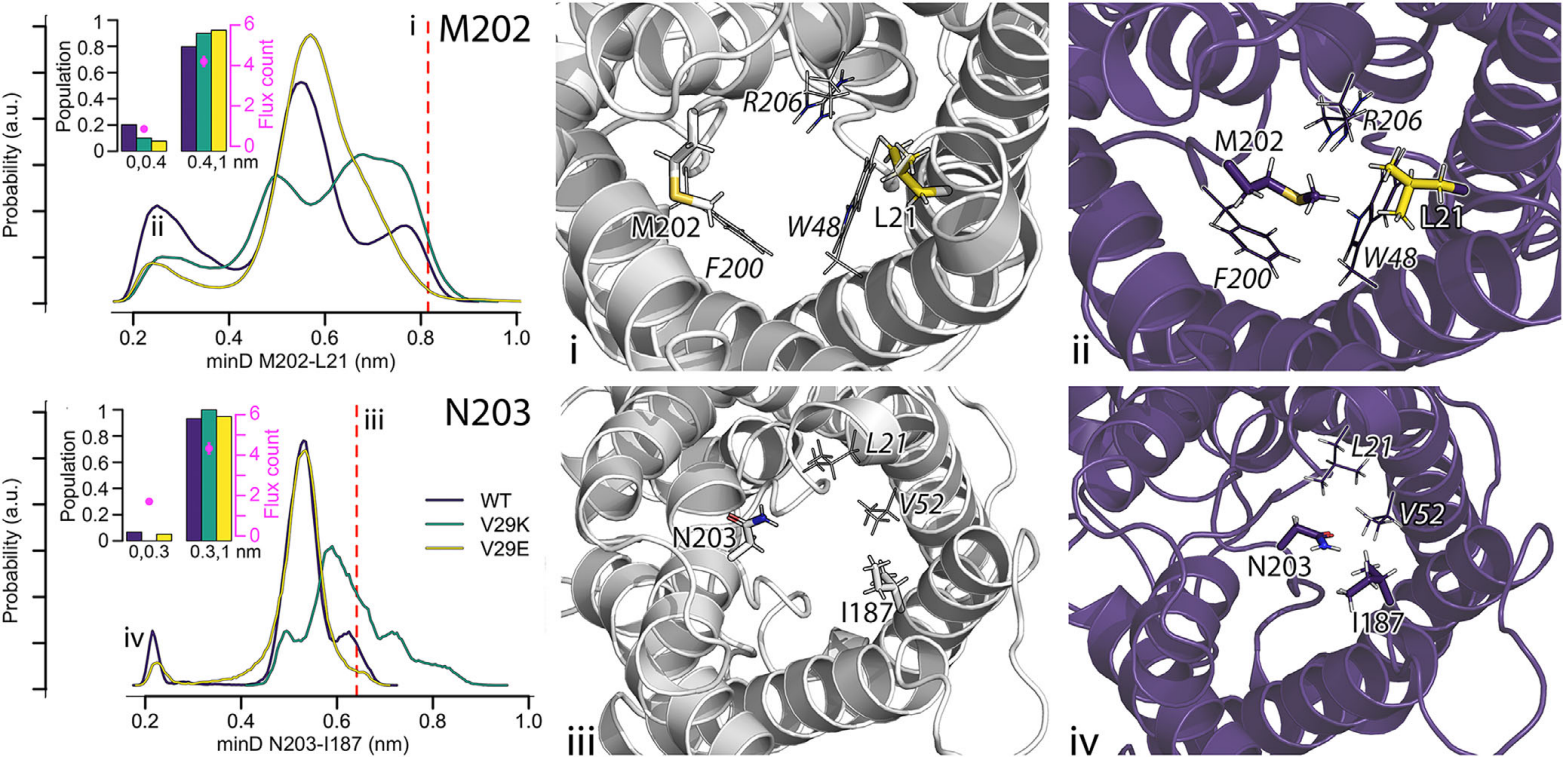
Positioning of M202^h2.‐2^ (top) and N203^h2.‐1^ (bottom) in the pore lumen, populations of the different positions and their water permeability. The movement of M202^h2.‐2^ into the pore lumen (top row) is described by measuring the minimal distance between its side chain and the side chain of L21^1.1^. The position of the corresponding residues in the crystal structure is shown in subfigure (i). Subfigure (ii) shows a close contact between the residues in wt GlpF. In subfigures (i) and (ii), L21^1.1^ is highlighted in yellow and shown in sticks; M202^h2.‐2^ is also shown as sticks. The selectivity filter residues in the direct neighborhood are shown as lines. The flipping of N203^h2.‐1^ (bottom row) into the pore lumen is characterized by the minimal distance of its side chain to the side chain of I187^5.1^. The orientation of the residues in the crystal structure is shown in subfigure (iii) and the orientation of N203^h2.‐1^ when flipped into the pore in a wt GlpF in subfigure (iv). In subfigures (iii) and (iv), N203^h2.‐1^ and I187^5.1^ are shown as sticks, while two other hydrophobic residues contributing to the pore narrowing/blockade L21^1.1^ and V52^2.1^ are shown as lines. Distances found in the crystal structure are highlighted by a red dashed lines.

Next to the F200^h2.‐4^ and A201^h2.‐3^ in L6, also M202^h2.‐2^and N203^h2.‐1^ can influence water passage (Figure 5). The often‐observed gating by M202^h2.‐2^ results from flipping of the side chain toward L21^1.1^. In a distance of more than 0.4 nm between the residues our Bayesian model predicts water permeabilities of 5.2 ± 0.3 water molecules per 5 ns, while shorter distances result in a reduction to 1.1 ± 0.1 water molecules each 5 ns. M202^h2.‐2^ is found within 0.4 nm to L21^1.1^ most often in the wt GlpF (20.2%), less often in V29K (10.1%) and least often in V29E (7.7%). For most of the simulation time, all proteins show shorter minimum distances between M202^h2.‐2^ and L21^1.1^ than the 0.82 nm observed in the crystal structure. Interestingly, the dynamics of M202^h2.‐2^ ranges from extremely floppy to persistent over hundreds of nanoseconds (Figure S14). To the best of our knowledge, only one mutational study attempted to mutate methionine in position h2.‐2, in detail, in *Pf*AQP L^h^ ^2.‐^ ^2^ was mutated either into M or V,^65^ even though methionine is found in the same position 8 times among the 20 resolved aqua(glycero)porin structures (in other structures leucine, isoleucine, and valine are present).^45^ Despite expression issues of L192M^h2.‐2^ in oocytes, water and glycerol permeability measurements indicate a probable minor contribution of position h2.‐2 on water/glycerol discrimination in *Pf*AQP.^65^ Our recent study shows that while M202^h2.‐2^ in *Ec*GlpF exhibits a gating behavior, M212^h2.‐2^ in *Hs*AQP4 does not.^45^ The latter, could be due to its direct interactions with the histidine in the ar/R filter, as mentioned in one recent study on *Hs*AQP4, however, without further analysis or speculations about the impact of this interaction. More is known about the amino acid in position of L21^1.1^ which was mutated in several studies. Presence of tyrosine in this position results in severe reduction of water permeability in lens AQP0, which is significantly improved upon its exchange for phenylalanine.^58^ The latter amino acid is found in this position in all other structurally resolved aquaporins except for AQPM, AQPZ, *Pf*AQP, and AQP10, which carry leucine/leucine or valine/leucine in the positions corresponding to M202^h2.‐2^/L21^1.1^ in *Ec*GlpF. Interestingly, the special structure of *Hs*AQP7 exhibits a reversed pair of these residues, that is, I^h2.‐2^ and M^1.1^.

The flexibility of loop L6 also allows N203^h2.‐1^ to occasionally translate into the pore in the direction of I187^5.1^ (Figure 5, bottom row). If N203^h2.‐1^ is closer to I187^5.1^ than 0.3 nm, the water permeability reduces from 4.3 ± 0.3 to 1.7 ± 0.1 water molecules per 5 ns. This is the case in 6.7% of simulation times analyzed for wt GlpF and 5.1% for the V29E mutant. For exemplarily time courses, see Figure S15.

**FIGURE 6:**
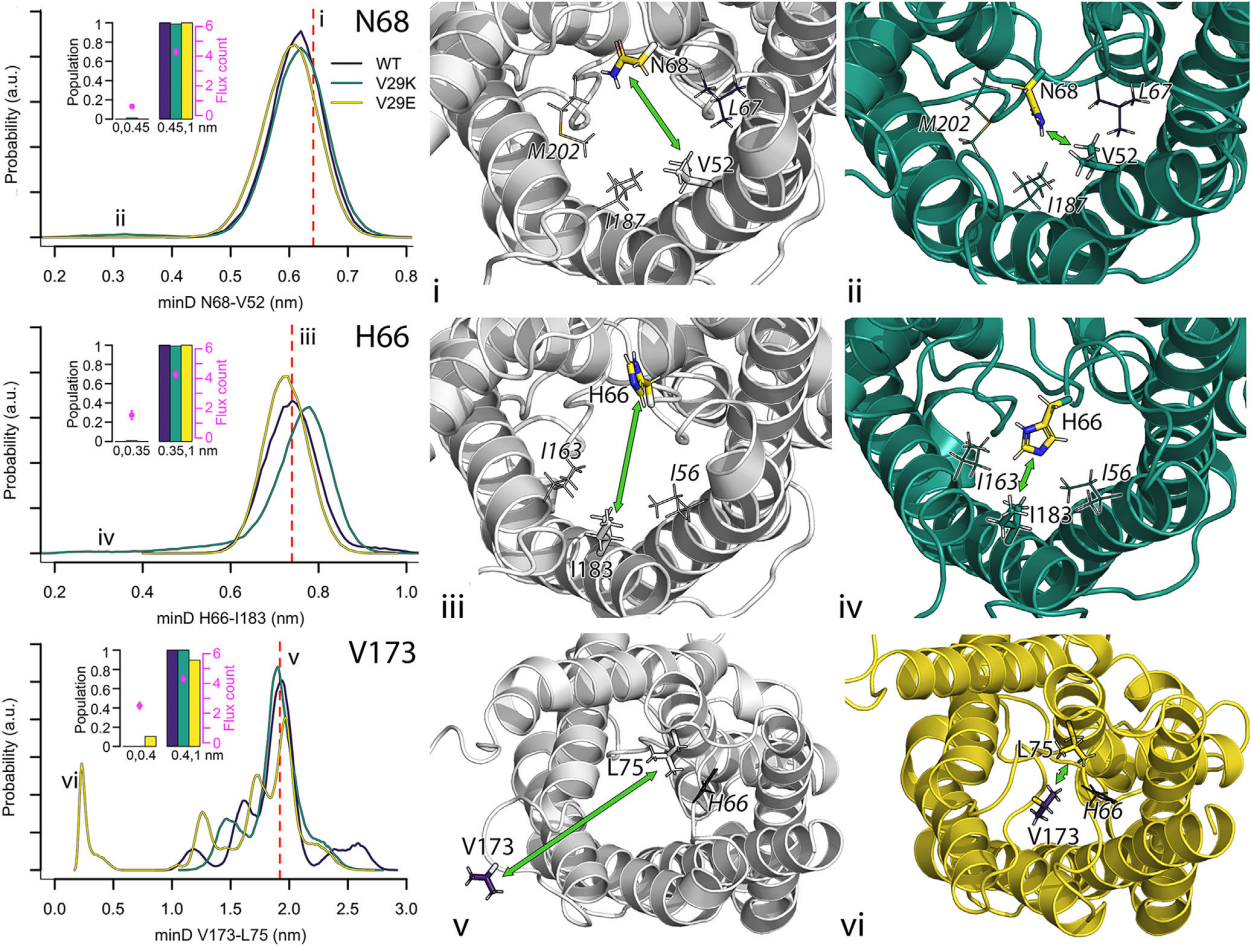
Orientational flexibility of N68^h1.‐1^, H66^h1.‐3^, and V173^4.17^, populations of the different positions and their water permeability. The movement of N68^h1.‐1^ into the pore (top row) is defined by measuring the minimal distance between its side chain and the side chain of V52^2.1^. The position of the residues in the crystal structure is shown in subfigure (i). Subfigure (ii) visualizes the shortened distance between the two residues (shown in sticks) in a V29K simulation. In both subfigures, N68^h1.‐1^ is highlighted in yellow and other residues forming the pore wall at the same pore cross section (L67^h1.‐2^, I187^5.1^, and M202^h2.‐2^) are shown as lines. The rarely observed flipping of H66^h1.‐3^ to the pore (middle row) is followed by measuring the minimal distance of its side chain and the side chain of I183^5.‐3^. The orientation of the residues in the crystal structure is shown in subfigure (iii) while subfigure (iv) highlights the movement of H66^h1.‐3^ toward I183^5.‐3^ (both shown as sticks, H66^h1.‐3^ is highlighted in yellow) in a V29K simulation. The direct neighbors I163^4.7^ and I56^2.5^ are shown as lines. The large movement of the fifth cytoplasmic loop (i.e., the loop connecting TM4 and TM5) to the entry lumen of the pore is characterized by measuring the minimal distance between the side chain of V173^4.17^ and L75^h1.6^. The orientation of the residues in the crystal structure is depicted in subfigure (v). Subfigure (vi) visualizes the orientation of V173^4.17^ close to L75^h1.6^ and H66^h1.‐3^ in a V29E simulation (yellow cartoon). In both subfigures, H66^h1.‐3^ is shown as lines, V173^4.17^ as purple sticks, and L75^h1.6^ as sticks. Distances found in the corresponding crystal structure are highlighted by a red dashed lines. The green arrows indicate the distances of interest.

Even less frequently, the other asparagine from the NPA filter (N68^h1.‐1^) can alter its orientation and stretch toward hydrophobic residues V52^2.1^, L67^h1.‐2^, and I187^5.1^, thus reducing the pore permeability from 4.3 ± 0.3 to 0.6 ± 0.1 water molecules per 5 ns (Figure 6, top row). This movement was analyzed by following the minimal distances between the side chains of N68^h1.‐1^ and V52^2.1^ over time. N68^h1.‐1^ is found in the pore if the distance drops under 0.45 nm. While such movement was never observed in wt GlpF, and it occurred rarely in V29E (two short flip events making up 0.04% of the analyzed simulation time), it was present in 1.3% of the simulation time analyzed for V29K where both short flipping events and a rather persistent switch were observed (Figure S16).

Additionally, we have observed flipping of H66^h1.‐3^, a conserved histidine in the cytosolic entry portal, to the pore in one V29K simulation for a total of 1.0% of the analyzed simulation time (Figure 6, mid row, Figure S17). The movement of histidine into the pore is characterized by its minimal distance to I183^5.‐3^. If this minimal distance becomes smaller than 0.35 nm, the histidine has flipped into the pore and the water permeability reduces from 4.2 ± 0.3 to 1.5 ± 0.3 water molecules per 5 ns. The histidine in this position has been suggested to (co)gate *Hs*AQP4,^45^, ^67^, ^68^ *Hs*AQP5,^43^ *Hs*AQP10,^69^ *Bt*AQP1,^45^ and *Ec*AQPZ^45^ in silico. Moreover, functional analysis of *Hs*AQP4^67^ and *h*AQP10^69^ and structural analysis on *Hs*AQP10^69^ defined this histidine residue as a possible pH gate. Remarkably, the function of several other AQPs including *Bt*AQP1^35^ were reported to not depend on pH even though this histidine is highly conserved (presence in 19 out of 20 nonredundant high‐resolution AQ(G)P structures) as we highlight in our recent study.^45^ Our previous in silico study suggested that the histidine flexibility and pH gating reported in previous research may result from differences in stabilizing H‐bonds of the respective histidine.^45^ In detail, we found stable H‐bonds and H^h1.‐3^ conformation in *Ec*GlpF, individual histidine flipping events and rather strong anchoring of H^h1.‐3^ in *Bt*AQP1 and *Ec*AQPZ, whereas the H^h1.‐3^ was very mobile in *Hs*AQP4, where hardly any H‐bonds with neighboring amino acid residues were formed.^45^ It is interesting to note that the average minimal distance between our mutated V29K and the H66^h1.‐3^ amounts to 1.9 ± 0.1 nm with 1.5 nm depicting their minimal distance. The fact that the mutation still influences the dynamics of residues so far apart highlights the complexity and cooperativity of protein residues in aquaporin pores, similarly to ligand induced conformational changes in a distant part of the protein.^70^, ^71^

An exception in the gating character is gating by V173^4.17^, measured as the minimum distance between V173^4.17^ and L75^h1.6^, which describes a large‐scale movement of the cytoplasmic loop 5 (also termed loop D in the literature) between TM4 and TM5 into the pore, which took place in one chain of one V29E simulation (Figure 6, Figure S18). In total, V173^4.17^ is within 0.4 nm to L75^h1.6^ in V29E for 10.4% of time and the close contact reduces water permeability from 4.3 ± 0.3 to 2.5 ± 0.2 water molecules per 5 ns. The same loop is known to gate plant aquaporins.^41^

Because the electrostatically driven orientation of water molecules in pores of aqua(glycero)porins assures for exclusion of the passage of charged molecules and ions,^15^, ^24^ we have investigated how the introduction of an additional positive (V29K) or negative (V29E) charge into the pore lumen alters the water orientation along the GlpF pore. Supplementary Figure S19 shows that the V29E mutation has only a minor impact on water orientation around R206^h2.2^. In the rest of the pore, the water is oriented as in wt GlpF. V29K, on the other hand, affects the preferred water orientation along the whole pore, with most significant changes in the vicinity of the ar/R filter. However, the changes in dipole orientation are still in line with the idea of a disrupted proton permeation via the Grotthuss mechanism.^72^ In contrast, the hydrogen bonding between pore residues and water is very similar for all three protein variants (Supplementary Figure S20). The only residue with strikingly different number of hydrogen bonds, non‐surprisingly, is the mutated V29^1.9^ (valine forms no hydrogen bonds, lysine 2.5 ± 0.1 and glutamic acid 4.8 ± 0.3 hydrogen bonds with water). Also, the hydrogen bonding of the nearby T137^4.23^ is slightly altered, with less hydrogen bonds in V29K (2.4 ± 0.1) and more hydrogen bonds in V29E (3.2 ± 0.2) compared to the wt (2.7 ± 0.1). Both residues are located in the periplasmic vestibule at the passage to the single‐file region.

## 3 CONCLUSION

**FIGURE 7:**
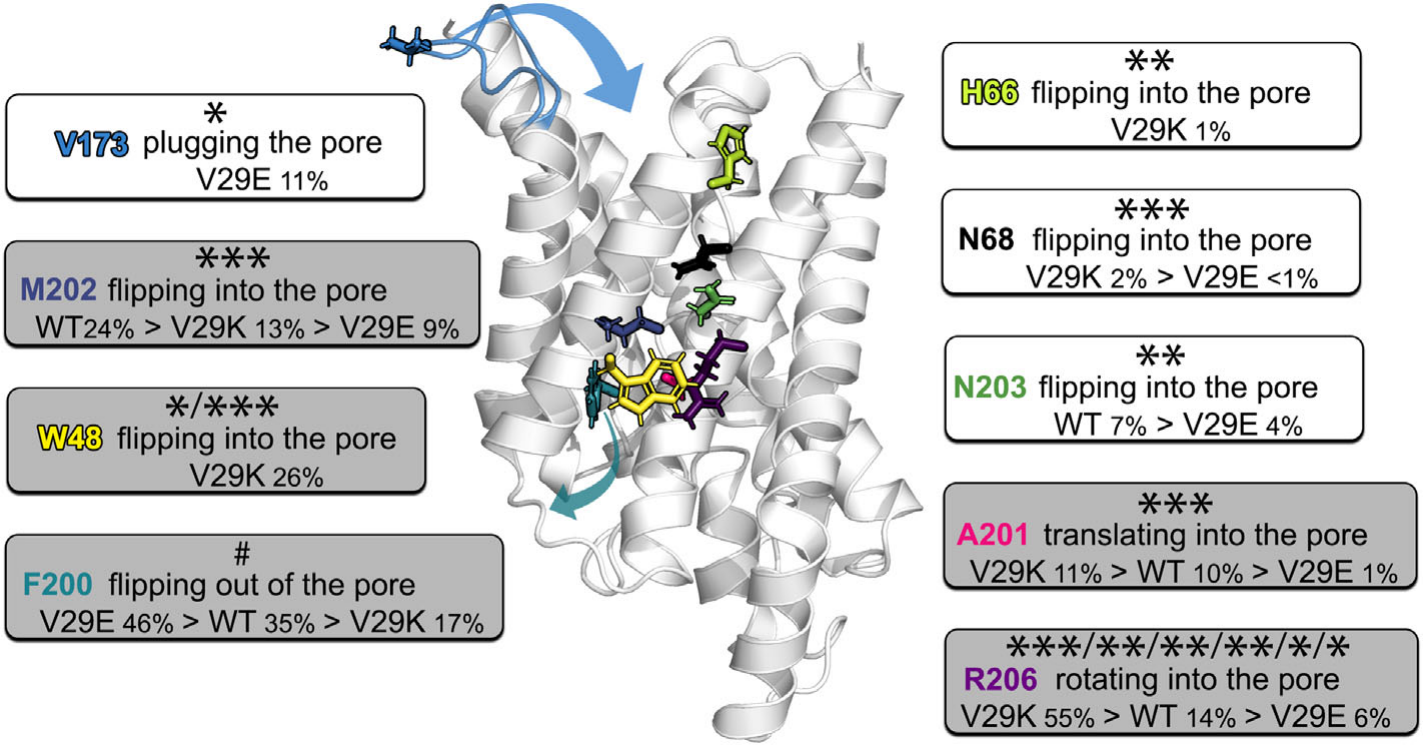
Summary of GlpF gating by pore lining residues. Residues found to flip into or from the pore during our 3‐μs‐long MD simulations are shown in sticks. Arrows indicate large‐scale movements of the corresponding residue. Compared to all other residues indicated here F200^h2.‐4^ has only a minor impact on water permeability. Fractions of populated states are indicated in percent of the total simulation time for V29E, the wild‐type (wt) GlpF, and V29K if applicable. The more often occurring gating motions are highlighted by gray background. The impact of the residue on the water permeability is indicated by stars: *60–100% water permeability, **30–59% water permeability, and ***0–29% water permeability as compared to the open state of the residue. For W48 and R206, multiple states are indicated. The ability of F200 to open the pore is pointed to by #.

We have successfully designed a mutation of the GlpF AQGP driving its water permeability by influencing the ar/R selectivity filter and tested its functionality both in silico and in vitro. Thereby, great agreement was obtained between MD and experiment on relative reduction and increase of water permeabilities for V29K and V29E mutants, respectively, relative to the wt protein. Along with our intention, the crystallized arginine position in the pore (open state) was stabilized in the V29E and strongly destabilized by the V29K mutant. However, the positioning of the arginine was not the sole determinant of water permeability. As our multi‐microsecond MD simulations show, multiple pore‐lining residues modulate the permeability of the channel (Figure 7). The complexity and cooperativity of their dynamics is emphasized by long‐range effects along the pore caused by our V29K^1.9^ mutation. In V29K, the W48^2.‐3^, another member of the ar/R selectivity filter obstructed the pore for a significant part of the simulation time. On the other hand, F200^h2.‐4^, the third member of the selectivity filter, most often slipped away from the narrow ar/R restriction site in V29E > wt GlpF > V29K, thus opening the pore. This opening was often counteracted by tight spacing between A201^h2.‐3^ and W48^2.‐3^ which reduced the water permeability in V29K, and less often also in the wt protein. The wild‐type pore was more often blocked by M202^h2.‐2^ and N203^h2.‐1^, compared to its mutant counterpart. Additionally, we have observed rare events including N68^h1.‐1^ flipping into the pore in V29K and V29E, H66^h1.‐3^ flipping into the pore in the cytoplasmic vestibule restricting the water permeability in V29K, and the cytoplasmic loop 5 plugging the pore in V29E. While N68^h1.‐1^‐mediated gating, to the best of our knowledge, was not reported in literature yet, the gating via the latter two are well known. The cytoplasmic histidine has been described to gate *Hs*AQP4,^45^, ^67^, ^68^ *Hs*AQP5,^43^ *Bt*AQP1,^45^ *Ec*AQPZ,^45^ and *Hs*AQP10.^69^ Loop 5 was shown to gate plant aquaporins.^41^

These results constitute the first experimental proof of modulation of water flux through aqua(glycero)porins by pore lining residues, including the conserved R^h2.2^ in the ar/R selectivity filter, under physiological conditions. Hence, channel gating by pore lining residues has to be considered a major modulator and determinant of water permeability through aqua(glycero)porins. While the major determinant of water permeability through the open pore are the number of H‐bonds single‐file water molecules may form with channel lining residues,^11^ the open probability of such a pore is modulated by flexible pore lining residues. This gating behavior is expected to be vastly different between AQPs as we have recently exemplified for *Bt*AQP1, *Hs*AQP4, *Ec*AQPZ^45^ and the herein studied *Ec*GlpF. It is expectable that the flexibility of pore lining residues can also influence the permeability of solutes through the narrow AQP pores and it could serve as one determinant of pore selectivity. Yet, the question of pore selectivity and specificity remains to be answered in future studies.

Taken together, this study provides novel structural insights into the gating mechanism of aquaporins. It thus paves the way for further investigations regarding the physiological implications of gating behavior and its importance for solute selectivity as well as the possibility of modulation by external stimuli like the transmembrane potential or lipid asymmetry. Moreover, it discloses a potential path to genetically engineer optimized aquaporin variants for biotechnological applications.

## 4 MATERIALS AND METHODS

### 4.1 Site directed mutagenesis

Coding regions for *Ec*GlpF were cloned as N‐terminal His‐tag fusion genes into pTrcHis vectors (Thermo Fisher Scientific). In addition, GlpF was modified by site‐directed mutagenesis to change the valine in position 29^1.9^ to glutamic acid (V29E) or lysine (V29K).

### 4.2 Protein expression, purification, labeling, and reconstitution

*Ec*GlpF overexpression, purification, labeling and reconstitution were performed as previously described.^11^, ^60^, ^61^, ^73^ pTrc plasmids were transformed into C43 (DE3) cells which were grown in lysogeny broth (LB) medium containing ampicillin overnight, diluted 40‐fold and grown until reaching an optical density of 0.6. Expression was induced by 1 mM isopropyl‐β‐d‐thiogalactopyranosid for 3 hr. Cells were harvested and pellets frozen at −80°C. Defrosted cell pellets were lysed, and the pelleted cell fraction was solubilized in a detergent‐containing (2% *n*‐octyl‐β‐d‐glucoside, OG) buffer. After the removal of the insoluble material by ultracentrifugation, the supernatant was further purified using affinity chromatography (Ni^2+^‐column). To reconstitute *Ec*GlpF wt and mutants into proteoliposomes (PLs) *E. coli* PLE (Avanti Polar Lipids) doped with 0.004 m% Atto633PPE was dried on a rotary evaporator, and rehydrated in Reco buffer (100 mM NaCl, 20 mM 3‐(*N*‐morpholino)propanesulfonic acid (MOPS), 1.4% OG, pH 7.4) to attain a final lipid concentration of 20 mg/ml. Subsequent to bath sonication, the clear suspension was incubated with equal amounts of protein diluted in Reco buffer at room temperature for an hour. Detergent was removed with stepwise addition of Biobeads SM‐2 (Bio‐Rad) within 36 hr. PLs were harvested by ultracentrifugation, resuspended, centrifuged to remove aggregates, and put through 21 extrusion cycles stacked with two polycarbonate filters with 100‐nm pore size using a mini‐extruder from Avanti Polar Lipids. This results in a unimodal radius distribution as seen from dynamic light scattering (DLS) measurements. Control vesicles were treated similarly. All samples were assayed without delay.

### 4.3 Relative water permeability estimation

PLs and control vesicles were mixed with equal amounts of hyperosmotic solution (300 mM sucrose, 100 mM NaCl, 20 mM MOPS, pH 7.4) in a stopped‐flow apparatus (SFM‐300, Bio‐Logic, Claix, France) at 4°C. As previously described,^9^, ^59^, ^74^, ^75^ we monitored the intensity of scattered light at 90° at a wavelength of 546 nm. To calculate water permeability values from light scattering, we used our recently found analytical solution.^11^, ^76^ To be able to estimate relative unitary water permeability values, the number of GlpF monomers per PL was counted using fluorescence correlation spectroscopy.^9^, ^11^, ^74^, ^77^ In brief, fluorescently labeled GlpF served to count fluorescent particles in a PL containing solution before and after micellation with detergent. The average radius of the respective vesicle populations was examined using DLS.

### 4.4 MD simulations

The structure of the tetrameric GlpF, containing residues T6‐E267 in each chain, was based on the crystal structure 1FX8.^18^ The missing atoms were added by MODELLER.^78^ MODELLER was also used to prolong the C‐terminus by residues 260‐PCDICVVE‐267. While the N‐terminus was modeled as a neutral amine, the C‐terminus was chosen to be negatively charged, as in the original sequence another glutamic acid follows immediately afterward. Histidine residues were singly protonated on N^ɛ^, except for H66^h1.‐3^ which carried the hydrogen on N^δ^, forming a hydrogen bond with the side chain oxygen of T72^h1.3^. All residues were in their standard protonation state at physiological pH. In an attempt to influence the positioning of R206^h2.2^ without introduction of mutations directly into the functional segments of the pore, V29^1.9^, located in the first transmembrane helix, opposite to R206^h2.2^ but shifted in the cytoplasmic direction, was mutated either to a negatively charged glutamic acid or positively charged lysine. The tetrameric proteins were then embedded in a simple symmetric model of the *E. coli* PLE membrane consisting of phosphatidylethanolamine (PE):phosphatidylglycerol (PG):cardiolipin in 67:23:10 molecular ratio, solvated by 70 waters per lipid and Na^+^ as counterions. Each PG carried a palmitoyl and a cyclopropanylated palmitoyl, while the tails of PE were palmitoyls and oleoyls. Each cardiolipin had a charge of −2e and the first palmitoyl lipid tail was cyclopropanylated in positions 9 and 10 and the other three tails were simple palmitoyls.

The simulation workflow followed our well established multiscaling procedure.^79^ In short, the energy minimized tetrameric wt GlpF was coarse‐grained using *martinize* and the Martini2 force field.^80^, ^81^ The lipids and the solvent were then added by *insane* ^82^ and the system was energy minimized in 5000 steps of steepest–descent energy minimization. Next, the system was equilibrated by performing a series of short MD simulation with applying position restrain on the protein and increasing simulation time steps (2, 5, 10, and 20 fs, for 10,000 steps each). Then, an equilibrated lipid distribution around the protein was assured for by a 1 μs long coarse‐grained simulation with applying position restrain on the backbone of the protein (for detailed information on the simulation settings, see^83^). The final frame was converted back to the atomistic resolution using *backward*,^84^ the CHARMM36(m) force field,^85^, ^86^, ^87^ and the TIP3P water model with Lennard–Jones parameters on hydrogen atoms as is typical for usage of TIP3P with the CHARMM force field.^88^ Then, the in vacuo energy minimized wt protein or the mutants were fitted as tetramers on the backmapped protein and the overlapping water molecules were removed. In order to avoid overlaps between the protein and the lipids, the system was energy minimized twice. In the first energy minimization of 10,000 steps, the protein was kept frozen. In the second energy minimization of 2000 steps, all atoms were allowed to move. Then, velocities were generated and the simulation system was equilibrated at 310 K by two position restrain simulations each lasting 5 ns. In the first one, the whole protein was position restrained, while in the second one, the position restrains on the protein side chains were relieved. In case of the mutants, in one replica, the side chain of the mutated residue 29^1.9^ was never restrained. The production run simulations were run at 310 K for 3 μs in two replicas for each protein variant (labeled wt GlpF, V29E, and V29K throughout the manuscript). All simulations were performed using GROMACS simulation engine version 5.^89^ To the applied atomistic simulation conditions belong the integration step of 2 fs, linear removal of the center of mass of the system each 100 steps, Nosé–Hoover thermostat^90^ with coupling constant of 0.5 ps, semi‐isotropic pressure coupling to 1 bar using the Parrinello–Rahman barostat,^91^ coupling constant of 5 ps, and compressibility of 4.5 × 10^−5^ bar^−1^. Bonds to hydrogens were constrained by LINCS,^92^ the electrostatics behind 1.2 nm was described by Paricle‐Mesh‐Ewald,^93^ and the van der Waals interactions were force‐switched between 0.8 and 1.2 nm using the Verlet cut‐off scheme.^94^ The trajectories of all atoms were saved every 10 ps in order to be able to track the individual water molecules passing the pores.

The analysis of the water flow through the pore of each chain was performed by the tool g_flux^95^ through a 2.2 nm long cylinder with 1.5 nm radius centered at the center of mass of each protein chain after the fit onto the crystal structure. The number of water molecules that passed the protein was corrected by a home‐written script for water molecules that were wrongly assigned as having passed the channel. The analysis of the other properties was carried out by standard GROMACS tools and home‐written scripts. The first 1 μs of each simulation was excluded from the analysis for equilibration purposes.

The images of the molecules were generated using PyMOL 2.5.0^96^ and the plots were done in R version 4.1.^97^

### 4.5 Bayesian data analysis

In order to elucidate the role of individual residues on the water flux, we fitted Bayesian generalized linear multilevel models^98^, ^99^ to our simulations. To obtain the data for these models, we binned simulations into 5‐ns intervals with 40% overlap between corresponding property and flux intervals, the former preceding the latter intervals (3 ns property alone, 2 ns overlap, and 3 ns flux alone). These choices were motivated by the observation that, on average, water molecules require 2.8, 3.8, and 2.6 ns to pass a wt GlpF, V29E, and V29K, respectively. Most of the transition events last less than 1 ns (47.3, 38.8, and 40.3%, for wt GlpF, V29E, and V29K, respectively) and 73.2, 75.2, and 69.8% less than 3 ns.

As outcome variable, we chose the number of water molecules passing the pore in a given interval, which constitutes a count variable without known upper bound. The negative‐binomial distribution with a log‐link, a generalization of the Poisson distribution that is common in such applications,^100^, ^101^ was chosen as data distribution (likelihood). As predicting variables, we used the percentage of each residue phase per binning interval. By definition, the residue phase percentages per residue sum to one such that the information on one of the phases is always redundant given all other phases. Accordingly, only *k* − 1 phase percentages per residue were considered where *k* is the total number of phases of that residue. Including all these predicting variables together in the same model allows us to estimate all effects of the indicated pore lining residues on the water permeability of the channel simultaneously, while considering the influence of all other residues. In addition, as the intervals are nested within pores, we added a varying intercept per pore (including four pores per GlpF type and two simulation replicas as well as the GlpF type) to account for the dependency of observations belonging to the same simulated pore.^99^ Prior distributions were chosen to be non‐ or weakly informative, thus having only negligible influence on the obtained inference in light of the amount of available data (599 time intervals, 2 simulation replicas, 3 GlpF types, and 4 chains, resulting in 14,376 data points).^98^

Bayesian statistical modeling was carried out in R^97^ using the brms package^98^, ^102^ and the Markov‐Chain Monte Carlo (MCMC) sampling tools of RStan.^103^, ^104^ Two independent MCMC chains were run each for 2000 iterations of which the first 1,000 were discarded as warmup, leading to a total of 2000 posterior samples used for inference. Convergence was checked using standard measures for MCMC convergence, namely Rhat and effective sample size measures.^99^, ^105^ Convergence was achieved for all models. The complete analysis scripts and results are available as supplementary material.

## AUTHOR CONTRIBUTIONS

**Valentin Schittny:** Investigation (supporting); writing – review and editing (supporting). **Paul‐Christian Bürkner:** Investigation (supporting); writing – original draft (supporting); writing – review and editing (supporting). **Christine Siligan:** Investigation (supporting); writing – review and editing (equal). **Andreas Horner:** Conceptualization (equal); funding acquisition (equal); investigation (equal); supervision (equal); visualization (supporting); writing – original draft (equal); writing – review and editing (equal). **Kristyna Pluhackova:** Conceptualization (equal); funding acquisition (equal); investigation (equal); supervision (equal); visualization (lead); writing – original draft (equal); writing – review and editing (equal).

## Supporting information


**FIGURE S1**. Generalized numbering scheme illustrated on a snake‐plot representation of *Ec*GlpF. White and black bold letters in darker circles represent the residue at the center of the membrane of the corresponding helix serving as a reference residue in the numbering scheme and are listed at the bottom for each helix. In addition, the highly‐conserved R206^h2.2^ and the mutated V29^1.9^ are depicted.
**FIGURE S2**. Average net flux through each wt GlpF and V29K or V29E pore each 100 ns, over the simulation time. Error bars are SEM over 8 proteins (i.e., 4 chains and 2 replica simulations).
**FIGURE S3**. Osmotic shrinkage of proteoliposomes. Representative stopped‐flow data of GlpF wt, V29E, and V29K (dots) reconstitution series and the corresponding analytical fits (dashed lines) to the data. Equal volumes of vesicle suspension and hyperosmotic solution (300 mM sucrose) were mixed at 4°C in 100 mM NaCl and 20 mM MOPS at pH 7.4. Dark violet data represents the empty control vesicles.
**FIGURE S4**. Quantification of membrane protein abundance in the lipid bilayer. Representative fluorescence correlation spectroscopy autocorrelation curves exemplarily shown for Atto488 labeled GlpF V29K containing (A) proteoliposomes and (B) micelles after the addition of 2% n‐octyl‐β‐d‐glucoside (OG) and 2% sodium dodecyl sulfate (SDS). Micellation leads to an increased number of particles per confocal volume as can be seen by the smaller autocorrelation amplitude in (B) and a faster diffusion time through the confocal volume due to their smaller size as compared to lipid vesicles in (A). The ratio of GlpF containing vesicles per confocal volume and micelles after detergent addition results in the average number (n_GlpF_) of GlpF monomers per proteoliposome (C). The buffer contained 100 mM NaCl and 20 mM MOPS at pH 7.4.
**FIGURE S5**. Root mean square deviations (RMSDs) of the tetrameric fold estimated for Cα atoms in the transmembrane helices and half helices (called here TM bundle and consisting or residues 7–35, 41–63, 69–79, 83–119, 145–167, 178–196, 204–217, 232–255) after the fit of the tetrameric TM bundle to the tetrameric crystal structure (1FX8). Two sets of simulations for wt GlpF (purple), V29K (green), and V29E (yellow) shown in the top and bottom plot.
**FIGURE S6**. Time course of the W48^2.‐3^ dihedral angle Cα‐Cβ‐Cγ‐ Cδ1 of V29K GlpF in individual chains in the two simulation replicas. The vertical dotted line separates the equilibration part of the simulation (first μs) and the production part of the simulation (1–3 μs). The red horizontal dashed line visualizes the W48^2.‐3^ dihedral angle in the crystal structure.
**FIGURE S7**. Time course of the minimal distance between sidechains of F200^h2.‐4^ and P196^5.10^ exemplarily shown for wt GlpF (top), and V29E (bottom). The movement of F200^h2.‐4^ toward P196^5.10^ can be of persistent nature (wt chain A, V29E chains C and D), of temporary nature (wt chain B), or floppy (V29E chain B). The vertical dotted line separates the equilibration part of the simulation (first μs) and the production part of the simulation (1–3 μs). The red horizontal dashed line visualizes the minimal distance in the crystal structure.
**FIGURE S8**. Time course of the minimal distance between sidechains of A201^h2.‐3^ and W48^2.‐3^ exemplarily shown for wt GlpF (top), V29K (middle), and V29E (bottom), one simulation each. The vertical dotted line separates the equilibration part of the simulation (first μs) and the production part of the simulation (1–3 μs). The red horizontal dashed line visualizes the minimal distance in the crystal structure.
**FIGURE S9**. 2D plot of minimal distances between the sidechains of A201^h2.‐3^ and W48^2.‐3^ versus F200^h2.‐4^ and P196^5.10^ of the wt GlpF, averaged over 100 ns time intervals in between 1 and 3 μs. The points are colored according to the average nominal flux in the same time intervals. The green triangle gives the minimal distances in the crystal structure.
**FIGURE S10**. 2D plot of minimal distances between the sidechains of A201^h2.‐3^ and W48^2.‐3^ versus F200^h2.‐4^ and P196^5.10^ of V29K, averaged over 100 ns time intervals in between 1 and 3 μs. The points are colored according to the average nominal flux in the same time intervals. The green triangle gives the minimal distances in the crystal structure.
**FIGURE S11**. 2D plot of minimal distances between the sidechains of A201^h2.‐3^ and W48^2.‐3^ versus F200^h2.‐4^ and P196^5.10^ of V29E, averaged over 100 ns time intervals in between 1 and 3 μs. The points are colored according to the average nominal flux in the same time intervals. The green triangle gives the minimal distances in the crystal structure.
**FIGURE S12**. Time course of the R206^h2.2^ dihedral angle Cα‐Cβ‐Cγ‐Cζ shown for one set of wt GlpF (top), V29K(middle), and V29E (bottom) simulations. The vertical dotted line separates the equilibration part of the simulation (first μs) and the production part of the simulation (1–3 μs). The red horizontal dashed lines visualize the dihedral angle and its two periodic images in the crystal structure.
**FIGURE S13**. Time course of the R206^h2.2^ dihedral angle Cα‐Cβ‐Cγ‐Cζ shown for a second set of wt GlpF (top), V29K(middle), and V29E (bottom) simulations. The vertical dotted line separates the equilibration part of the simulation (first μs) and the production part of the simulation (1–3 μs). The red horizontal dashed lines visualize the dihedral angle and its two periodic images in the crystal structure.
**FIGURE S14**. Time course of the minimal distance between sidechains of M202^h2.‐2^ and L21^1.1^ exemplarily shown for wt GlpF (top), V29K (middle), and V29E (bottom), one simulation each. The vertical dotted line separates the equilibration part of the simulation (first μs) and the production part of the simulation (1–3 μs). The red horizontal dashed line visualizes the minimal distance in the crystal structure.
**FIGURE S15**. Time course of the minimal distance between sidechains of N203^h2.‐1^ and I187^5.1^ exemplarily shown for wt GlpF (top) and V29E (bottom), one simulation each. The vertical dotted line separates the equilibration part of the simulation (first μs) and the production part of the simulation (1–3 μs). The red horizontal dashed line visualizes the minimal distance in the crystal structure.
**FIGURE S16**. Time course of the minimal distance between sidechains of N68^h1.‐1^ and V52^2.1^ exemplarily shown for V29K (top) and V29E (bottom). The movement can be of persistent nature (V29K chain D), or floppy nature (V29E chain D, V29K chain D). The vertical dotted line separates the equilibration part of the simulation (first μs) and the production part of the simulation (1–3 μs). The red horizontal dashed line visualizes the minimal distance in the crystal structure.
**FIGURE S17**. Time course of the minimal distance between sidechains of H66^h1.‐3^ and I183^5.‐3^ exemplarily shown for V29K. The vertical dotted line separates the equilibration part of the simulation (first μs) and the production part of the simulation (1–3 μs). The red horizontal dashed line visualizes the minimal distance in the crystal structure.
**FIGURE S18**. Time course of the minimal distance between sidechains of V173^4.17^ and L75^h1.6^ shown for one V29E simulation, where loop D of chain B moved into the pore lumen. The vertical dotted line separates the equilibration part of the simulation (first μs) and the production part of the simulation (1–3 μs). The red horizontal dashed line visualizes the minimal distance in the crystal structure.
**FIGURE S19**. Orientation of the dipoles of water molecules passing the pore of wt GlpF and the mutants. The orientation is described as the tilt of the water dipole to the membrane normal. On the top the localization of important residues (i.e., the mutated residue 29^1.9^, R206^h2.2^ in the ar/R filter and N68^h1.‐1^ and N203^h2.‐1^) from the NPA filters are indicated.
**FIGURE S20**. Number of hydrogen bonds between pore lining residues and water. The residue T137^4.23^ is found in the vicinity of residue 29^1.9^. The barplot shows the average and SEM of the average number of hydrogen bonds (H‐bonds) in 1‐3 μs of the simulation time in each channel and simulations. VKE29 stands for V29 (WT), K29 (V29K mutant) and E29 (V29E mutant).

## Data Availability

The data that support the findings of this study are available from the corresponding author upon reasonable request.
