# Supplementary material for: Multiple pore lining residues modulate water permeability of GlpF
Source: Protein Sci. 2022 Sep 21;31(10):e4431. doi: 10.1002/pro.4431 (PMC9490802; doi:10.1002/pro.4431)
Supplement: Supplementary file 1 — FIGURE S1. Generalized numbering scheme illustrated on a snake‐plot representation of EcGlpF. White and black bold letters in darker circles represent the residue at the center of the membrane of the corresponding helix serving as a reference residue in the numbering scheme and are listed at the bottom for each helix. In addition, the highly‐conserved R206h2.2 and the mutated V291.9 are depicted. FIGURE S2. Average net flux through each wt GlpF and V29K or V29E pore each 100 ns, over the simulation time. Error bars are SEM over 8 proteins (i.e., 4 chains and 2 replica simulations). FIGURE S3. Osmotic shrinkage of proteoliposomes. Representative stopped‐flow data of GlpF wt, V29E, and V29K (dots) reconstitution series and the corresponding analytical fits (dashed lines) to the data. Equal volumes of vesicle suspension and hyperosmotic solution (300 mM sucrose) were mixed at 4°C in 100 mM NaCl and 20 mM MOPS at pH 7.4. Dark violet data represents the empty control vesicles. FIGURE S4. Quantification of membrane protein abundance in the lipid bilayer. Representative fluorescence correlation spectroscopy autocorrelation curves exemplarily shown for Atto488 labeled GlpF V29K containing (A) proteoliposomes and (B) micelles after the addition of 2% n‐octyl‐β‐d‐glucoside (OG) and 2% sodium dodecyl sulfate (SDS). Micellation leads to an increased number of particles per confocal volume as can be seen by the smaller autocorrelation amplitude in (B) and a faster diffusion time through the confocal volume due to their smaller size as compared to lipid vesicles in (A). The ratio of GlpF containing vesicles per confocal volume and micelles after detergent addition results in the average number (nGlpF) of GlpF monomers per proteoliposome (C). The buffer contained 100 mM NaCl and 20 mM MOPS at pH 7.4. FIGURE S5. Root mean square deviations (RMSDs) of the tetrameric fold estimated for Cα atoms in the transmembrane helices and half helices (called here TM bundle and consisting or res [file PRO-31-e4431-s001.docx]

***Supplementary Information:***

**Multiple Pore Lining Residues modulate Water Permeability of GlpF**

Kristyna Pluhackova^1#^, Valentin Schittny^2^, Paul-Christian Bürkner^1^, Christine Siligan^3^, Andreas Horner^3#^

^1^ Stuttgart Center for Simulation Science, Cluster of Excellence EXC 2075, University of Stuttgart, Universitätstr. 32, 70569 Stuttgart, Stuttgart, Germany;

^2^ Department of Biosystems Science and Engineering, Eidgenössische Technische Hochschule (ETH) Zurich, Mattenstr. 26, 4058 Basel, Switzerland

^3^ Institute of Biophysics, Johannes Kepler University, Gruberstr. 40, 4020 Linz, Austria

^#^Correspondence to: Andreas Horner, [andreas.horner@jku.at](mailto:andreas.horner@jku.at)
Kristyna Pluhackova, [kristyna.pluhackova@simtech.uni-stuttgart.de](mailto:kristyna.pluhackova@simtech.uni-stuttgart.de)

*
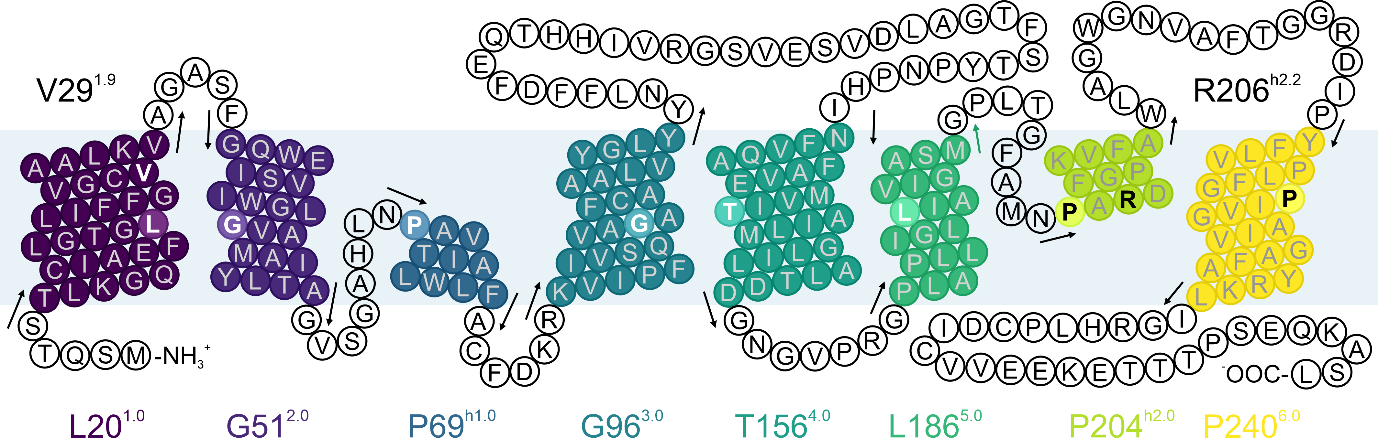
*

***Figure S1. Generalized numbering scheme illustrated on a snake-plot representation of EcGlpF.*** *White and black bold letters in darker circles represent the residue at the center of the membrane of the corresponding helix serving as a reference residue in the numbering scheme and are listed at the bottom for each helix. In addition, the highly-conserved R206^h2.2^ and the mutated V29^1.9^ are depicted.*

*
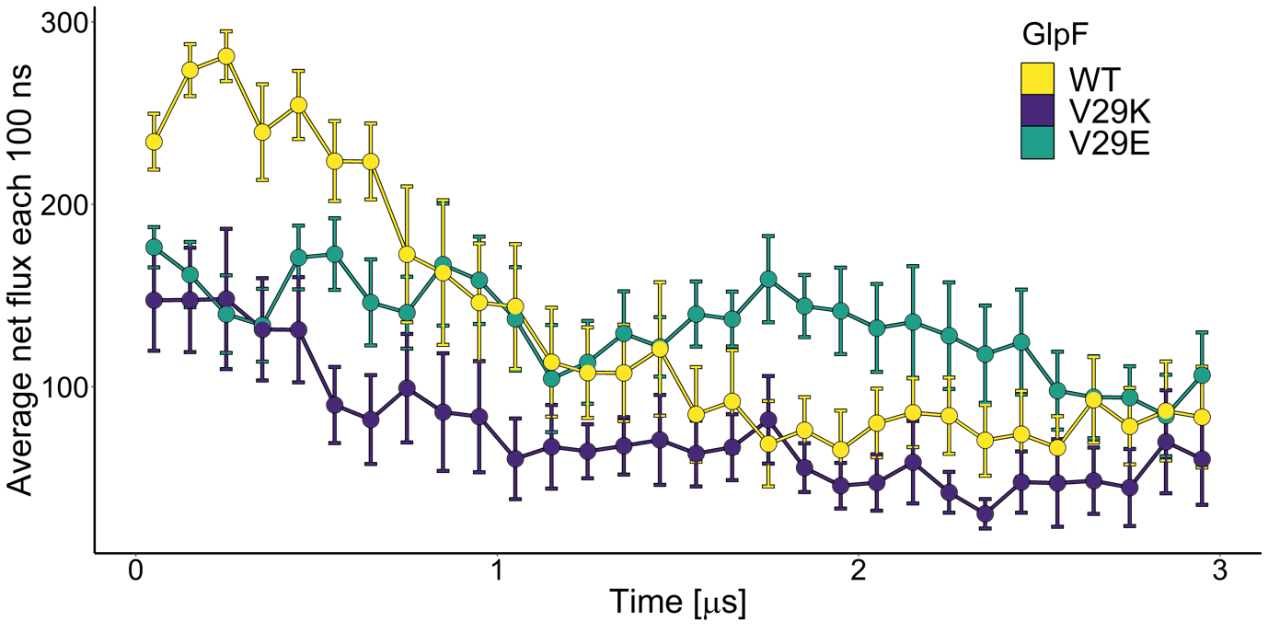
*

**Figure S2. Average net flux** through each wt GlpF and V29K or V29E pore each 100 ns, over the simulation time. Error bars are SEM over 8 proteins (i.e. 4 chains and 2 replica simulations).

*
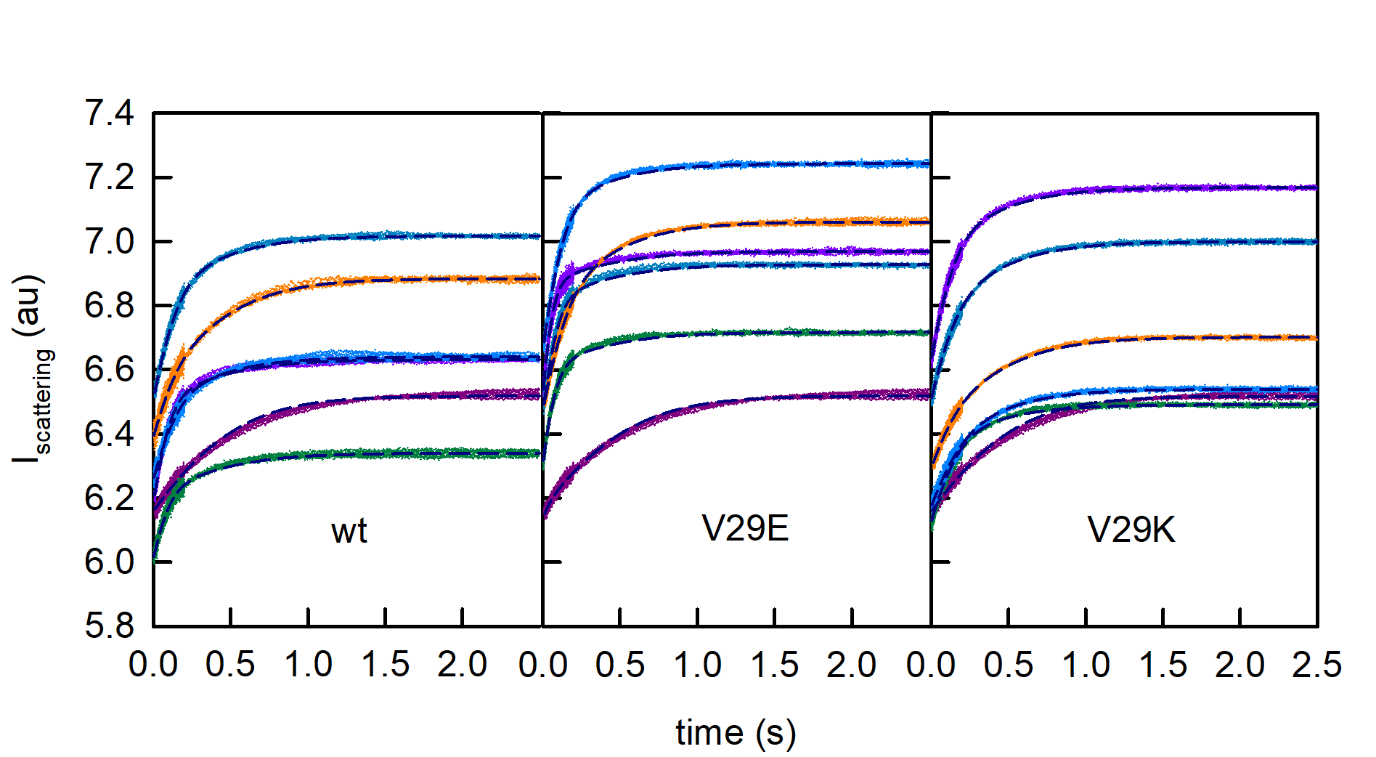
*

***Figure S3. Osmotic shrinkage of proteoliposomes.*** *Representative stopped-flow data of GlpF wt, V29E, and V29K (dots) reconstitution series and the corresponding analytical fits (dashed lines) to the data. Equal volumes of vesicle suspension and hyperosmotic solution (300 mM sucrose) were mixed at 4°C in 100mM NaCl and 20mM MOPS at pH 7.4. Dark violet data represents the empty control vesicles.*

|  |  |
| --- | --- |

*
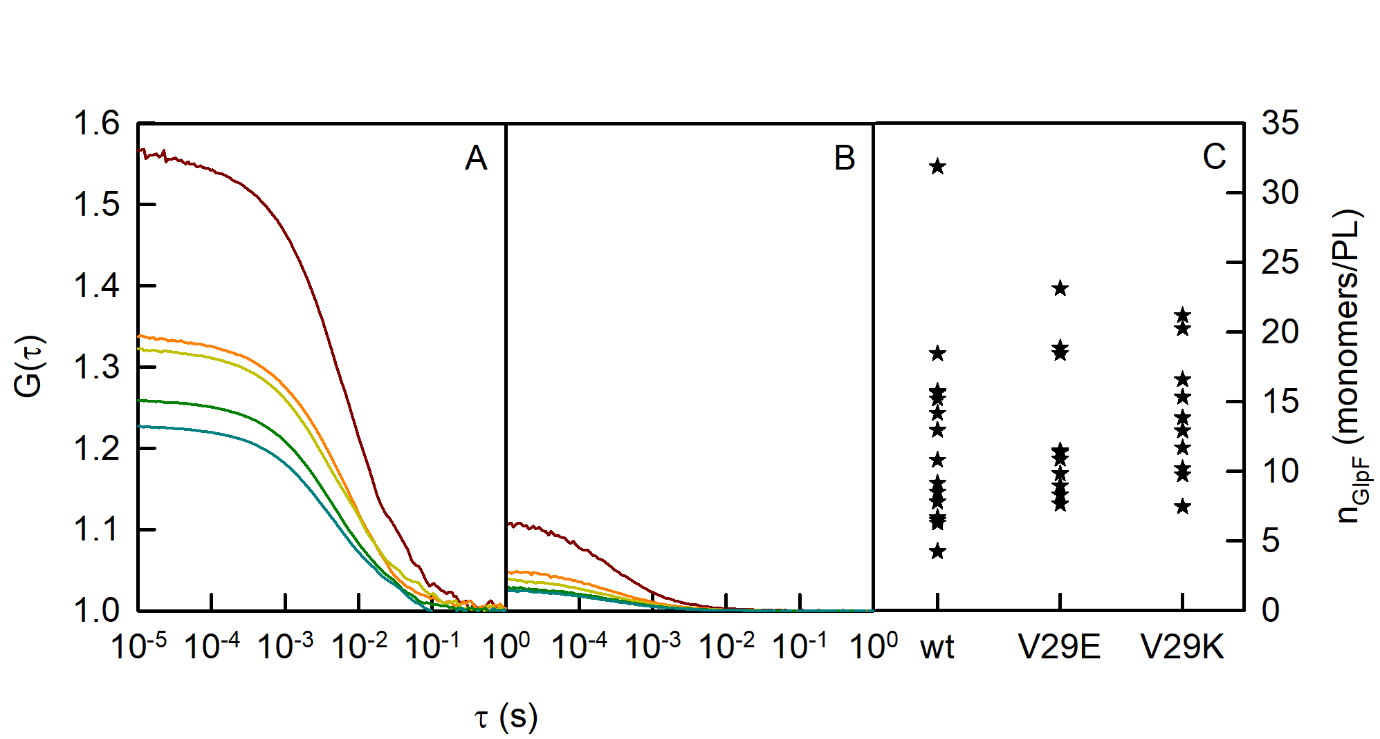
*

***Figure S4.*** ***Quantification of membrane protein abundance in the lipid bilayer.*** *Representative fluorescence correlation spectroscopy autocorrelation curves exemplarily shown for Atto488 labeled GlpF V29K containing (A) proteoliposomes and (B) micelles after the addition of 2% n-octyl-β-d-glucoside (OG) and 2% sodium dodecyl sulfate (SDS). Micellation leads to an increased number of particles per confocal volume as can be seen by the smaller autocorrelation amplitude in (B) and a faster diffusion time through the confocal volume due to their smaller size as compared to lipid vesicles in (A). The ratio of GlpF containing vesicles per confocal volume and micelles after detergent addition results in the average number (n_GlpF_) of GlpF monomers per proteoliposome (C). The buffer contained 100mM NaCl and 20mM MOPS at pH 7.4.*


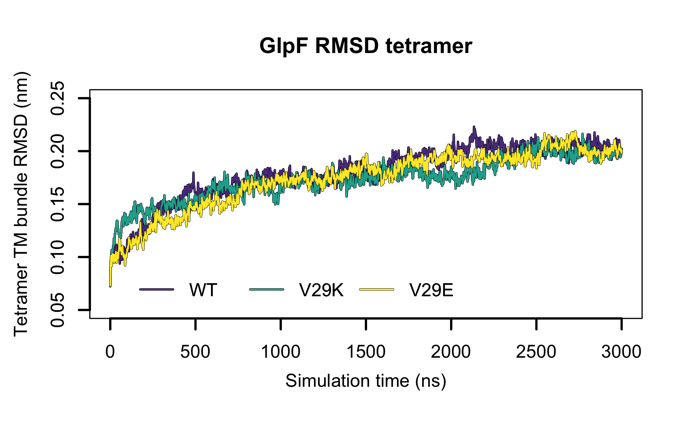

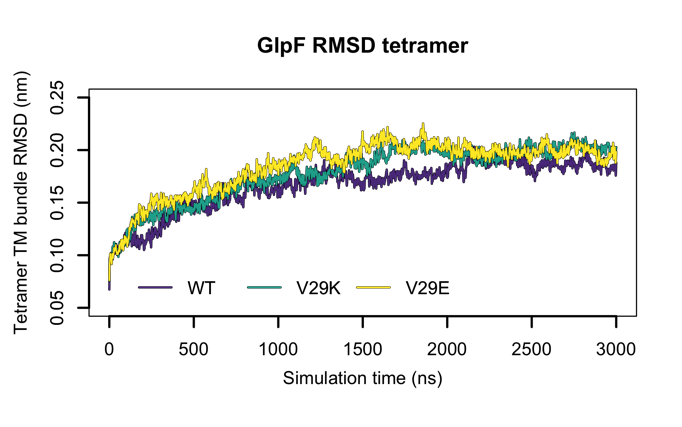


***Figure S5. Root mean square deviations (RMSDs) of the tetrameric fold*** *estimated for Cα atoms in the transmembrane helices and half helices (called here TM bundle and consisting or residues 7-35, 41-63, 69-79, 83-119, 145-167, 178-196, 204-217, 232-255) after the fit of the tetrameric TM bundle to the tetrameric crystal structure (1FX8). Two sets of simulations for wt GlpF (purple), V29K (green), and V29E (yellow) shown in the top and bottom plot.*


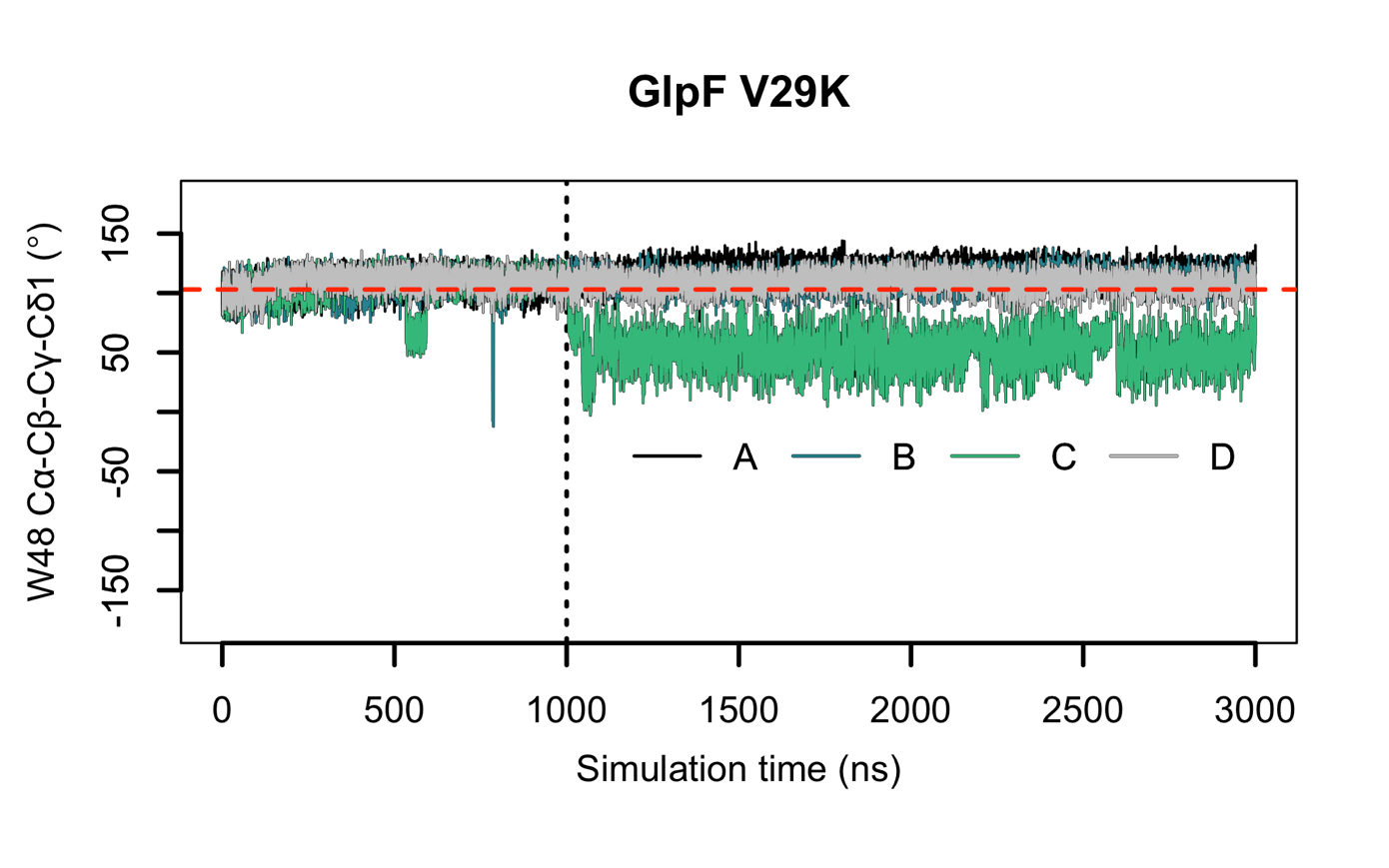

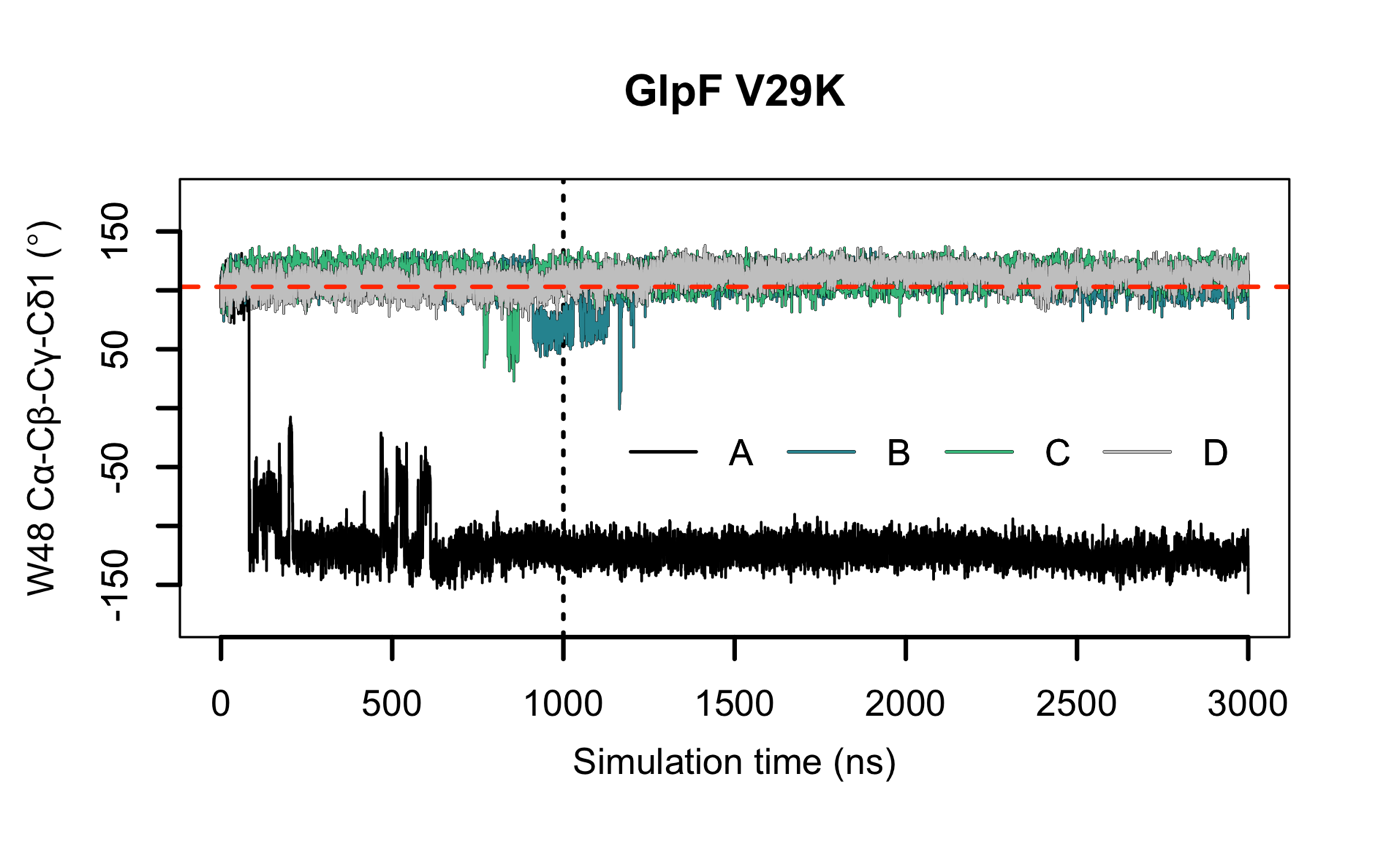


**Figure S6. Time course of the W48^2.-3^ dihedral angle** Cα-Cβ-Cγ- Cδ1 of V29K GlpF in individual chains in the two simulation replicas. The vertical dotted line separates the equilibration part of the simulation (first µs) and the production part of the simulation (1-3 µs). The red horizontal dashed line visualizes the W48^2.-3^ dihedral angle in the crystal structure.

*
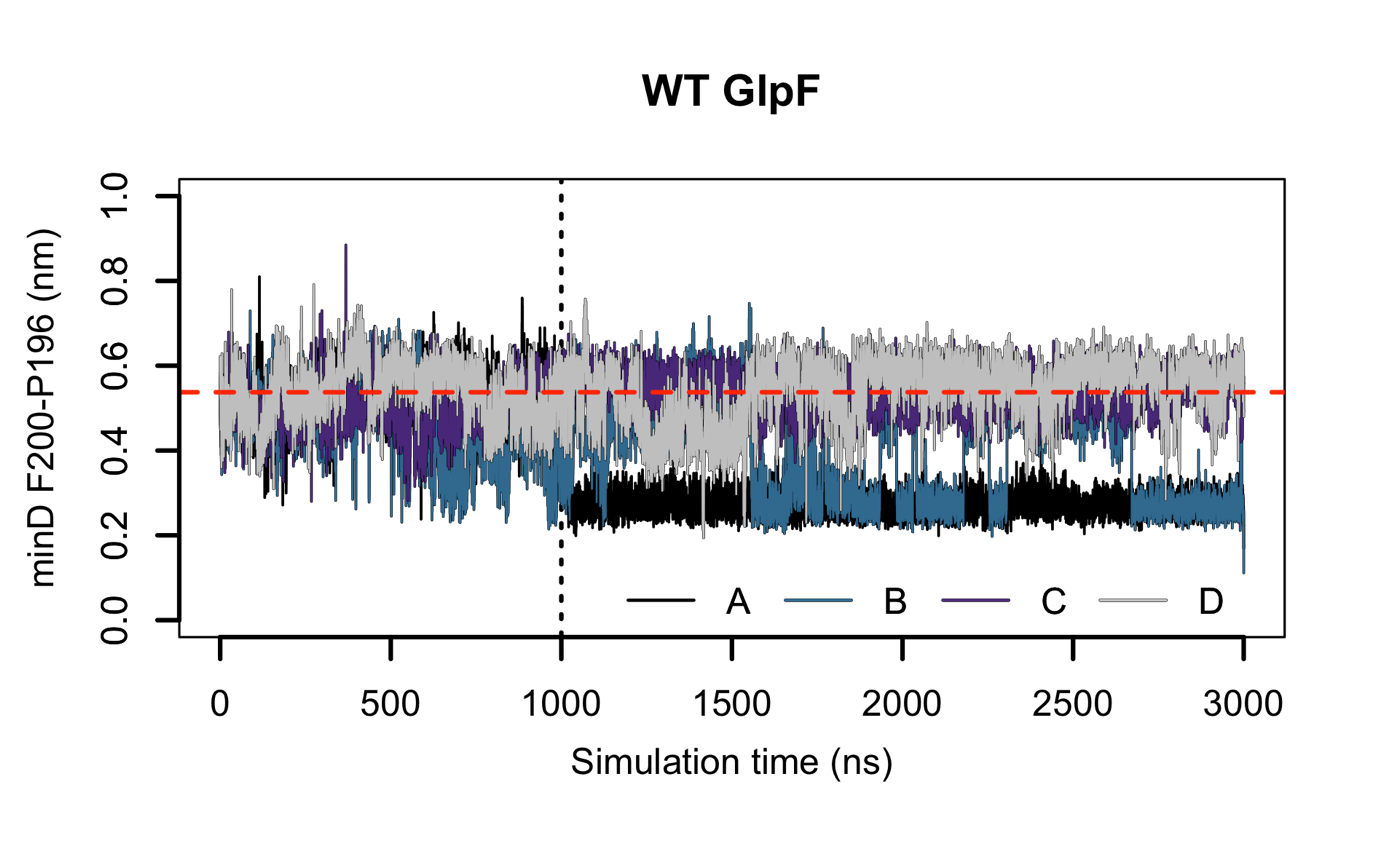

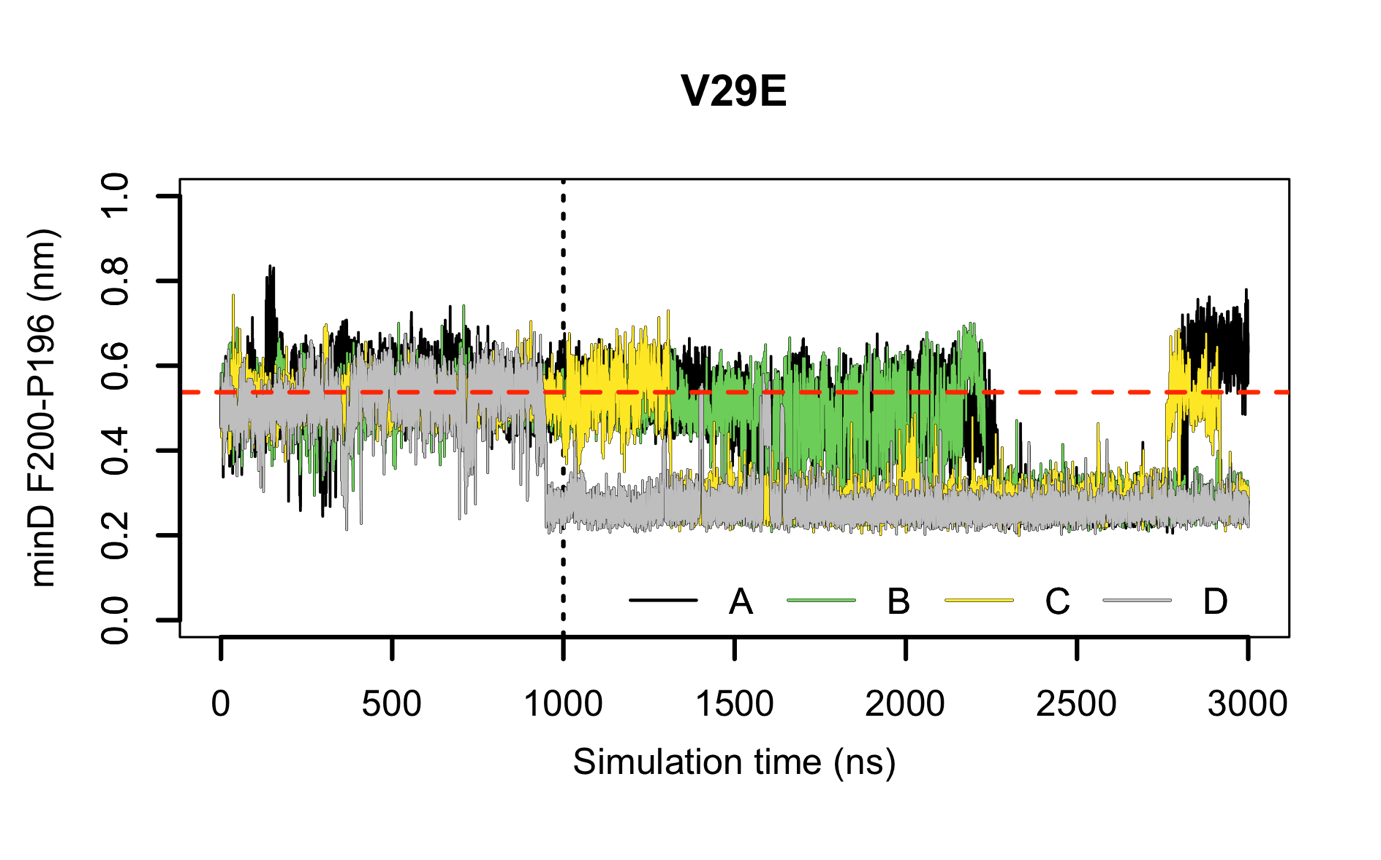
*

**Figure S7. Time course of the minimal distance between sidechains of F200^h2.-4^ and P196^5.10^** exemplarily shown for wt GlpF (top), and V29E (bottom). The movement of F200^h2.-4^ towards P196^5.10^ can be of persistent nature (wt chain A, V29E chains C and D), of temporary nature (wt chain B), or floppy (V29E chain B). The vertical dotted line separates the equilibration part of the simulation (first µs) and the production part of the simulation (1-3 µs). The red horizontal dashed line visualizes the minimal distance in the crystal structure.


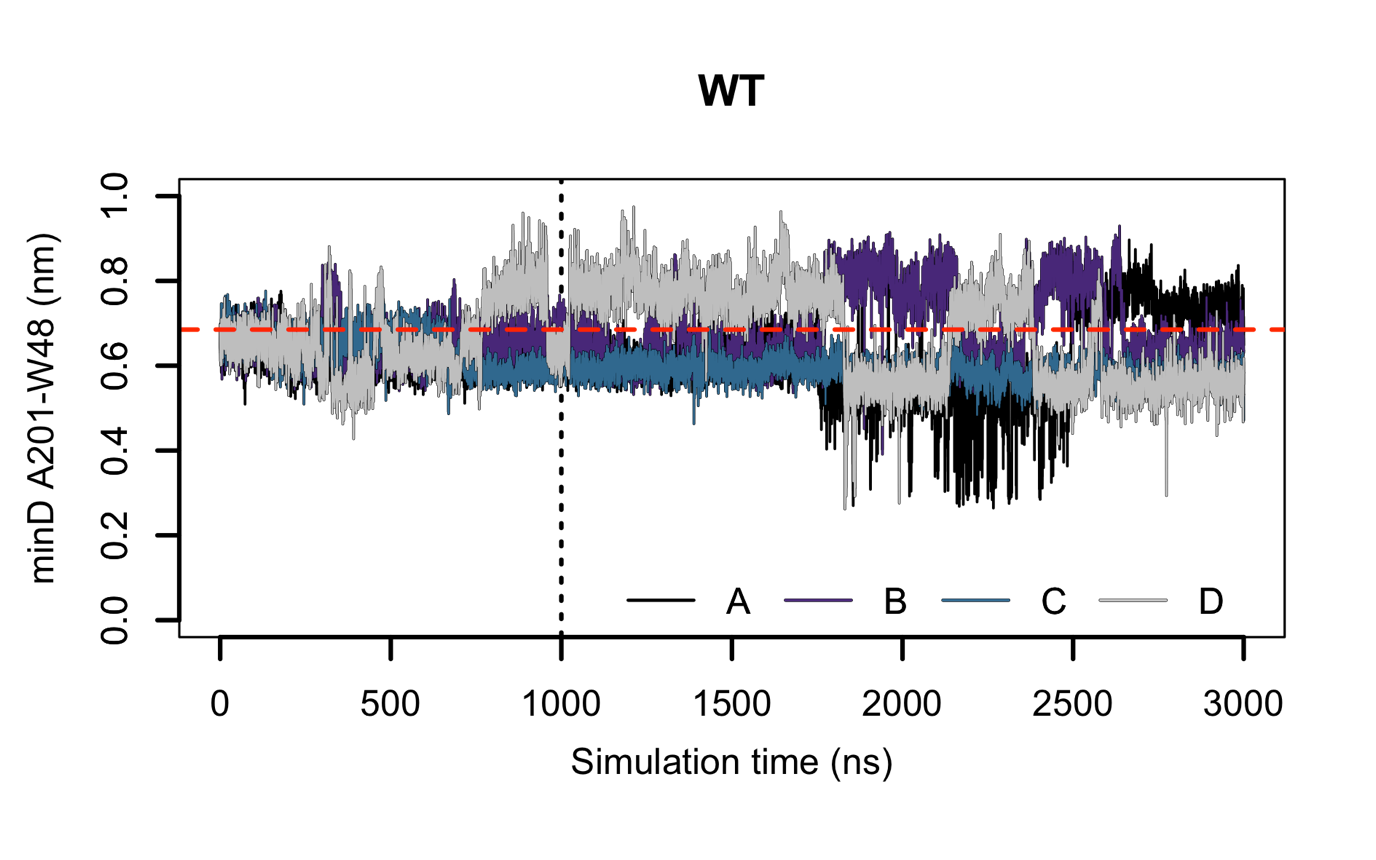

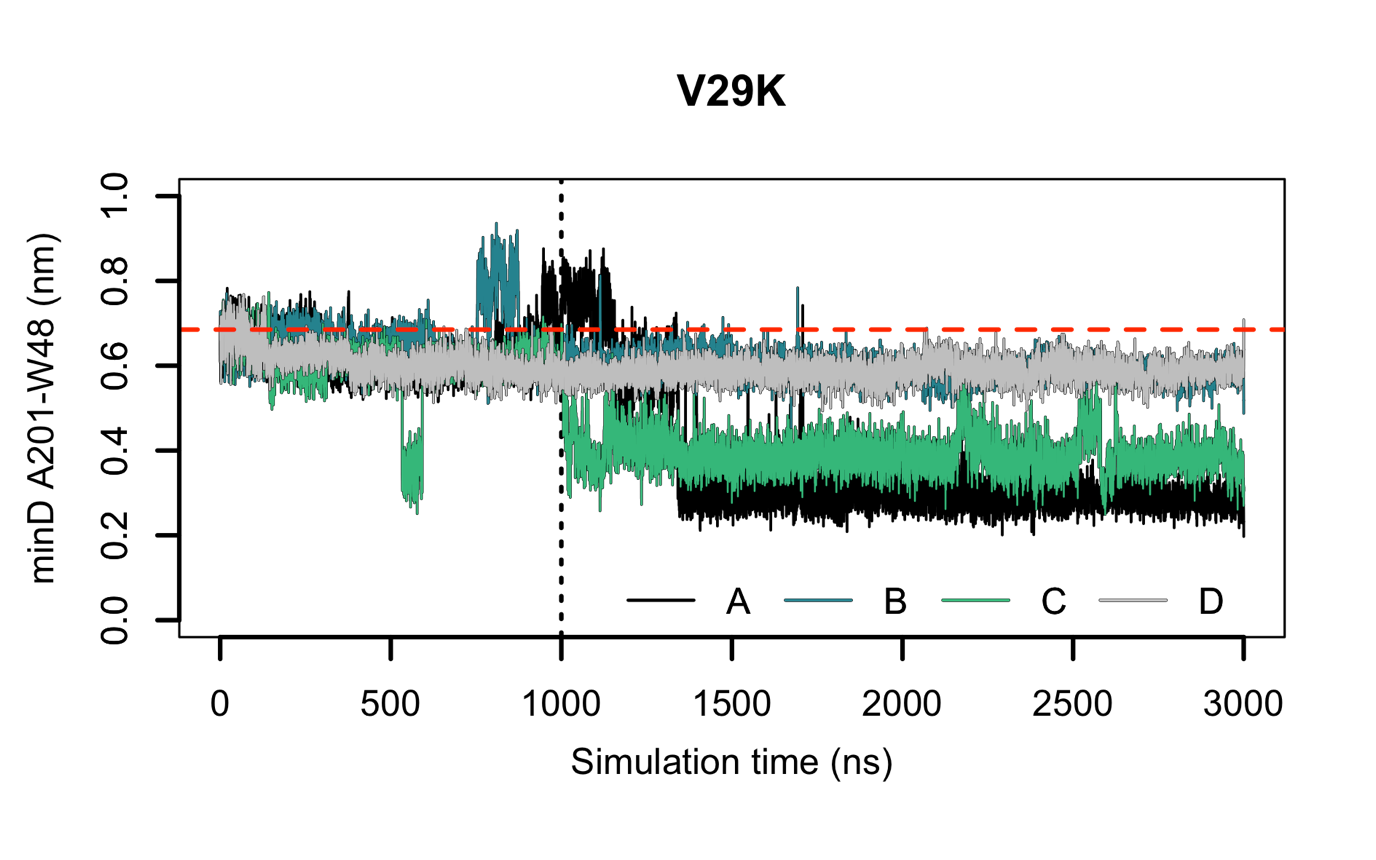

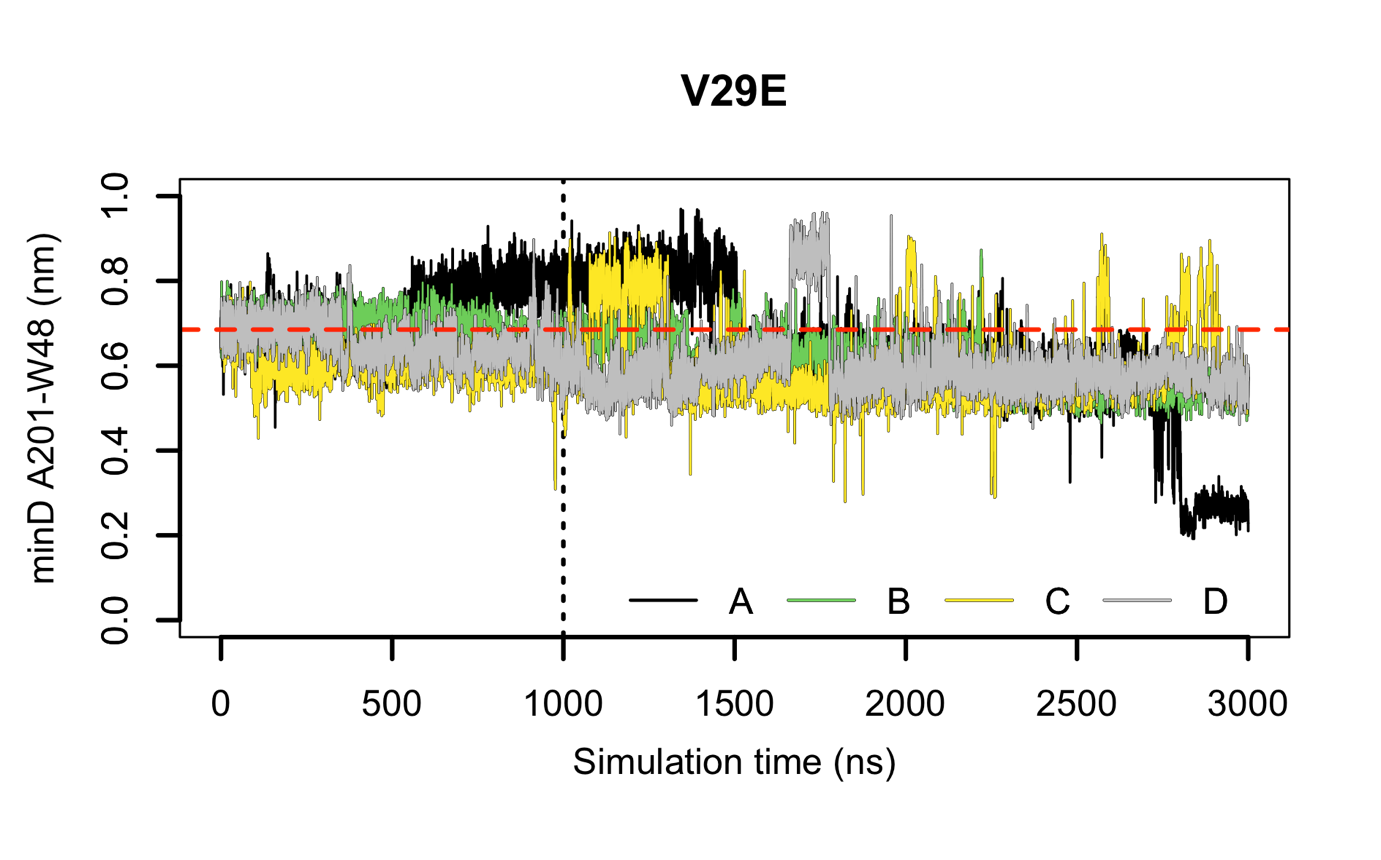


**Figure S8. Time course of the minimal distance between sidechains of A201^h2.-3^ and W48^2.-3^** exemplarily shown for wt GlpF (top), V29K (middle), and V29E (bottom), one simulation each. The vertical dotted line separates the equilibration part of the simulation (first µs) and the production part of the simulation (1-3 µs). The red horizontal dashed line visualizes the minimal distance in the crystal structure.

| 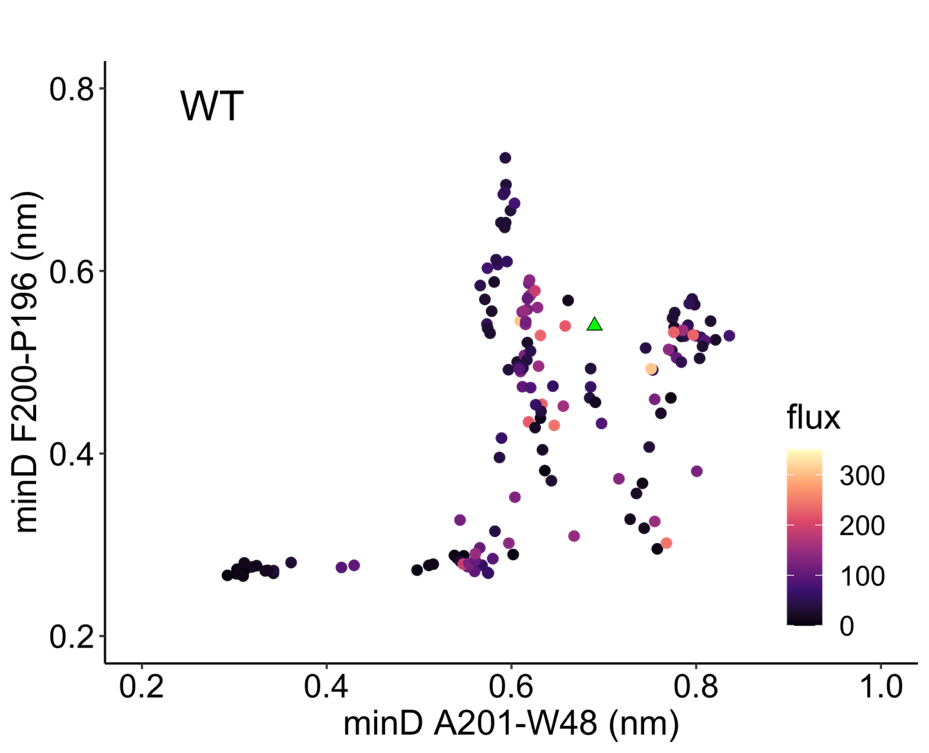 | **Figure S9.** **2D plot of minimal distances between the sidechains of A201^h2.-3^ and W48^2.-3^ versus F200^h2.-4^ and P196^5.10^ of the wt GlpF**, averaged over 100 ns time intervals in between 1 and 3 µs. The points are colored according to the average nominal flux in the same time intervals. The green triangle gives the minimal distances in the crystal structure. |
| --- | --- |
| 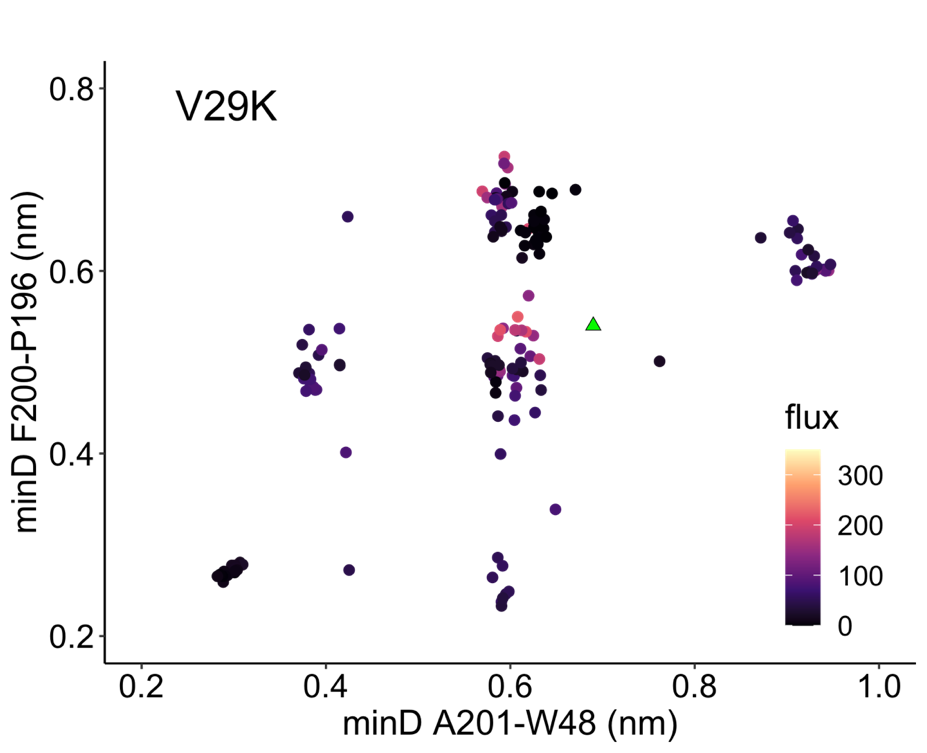 | **Figure S10. 2D plot of minimal distances between the sidechains of A201^h2.-3^ and W48^2.-3^ versus F200^h2.-4^ and P196^5.10^ of V29K**, averaged over 100 ns time intervals in between 1 and 3 µs. The points are colored according to the average nominal flux in the same time intervals. The green triangle gives the minimal distances in the crystal structure. |
| 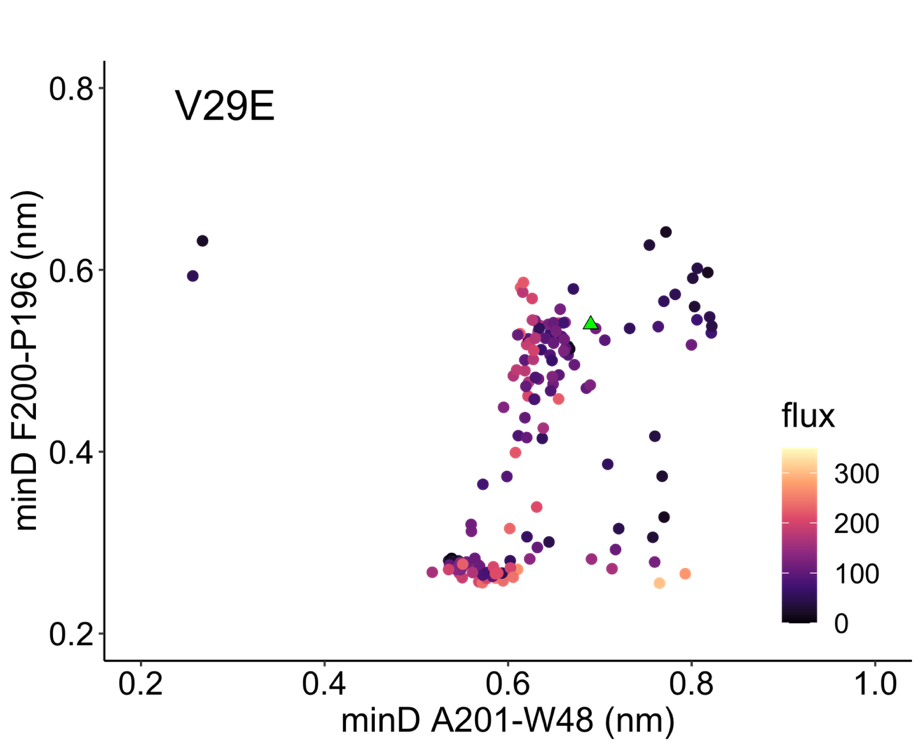 | **Figure S11. 2D plot of minimal distances between the sidechains of A201^h2.-3^ and W48^2.-3^ versus F200^h2.-4^ and P196^5.10^ of V29E**, averaged over 100 ns time intervals in between 1 and 3 µs. The points are colored according to the average nominal flux in the same time intervals. The green triangle gives the minimal distances in the crystal structure. |


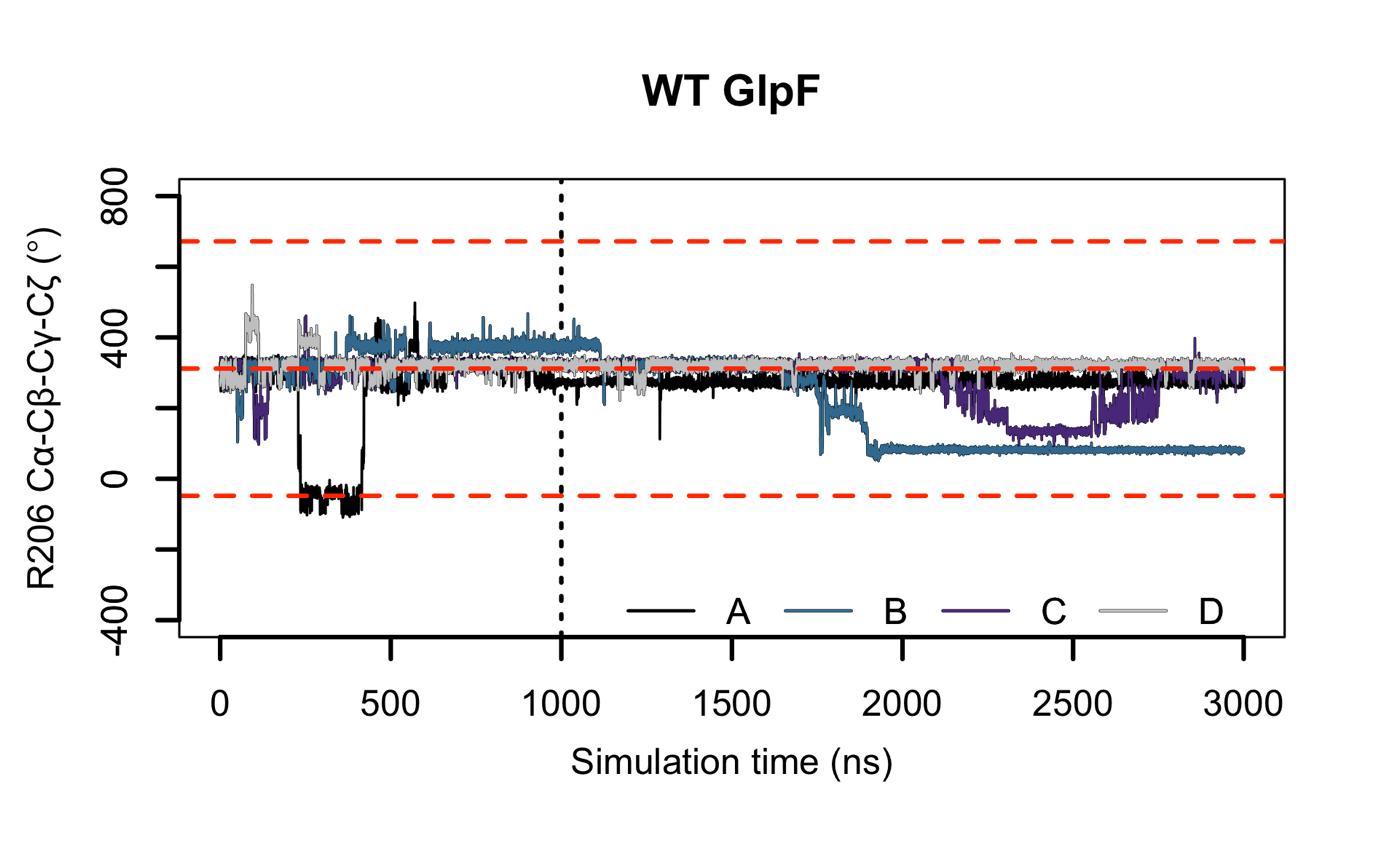


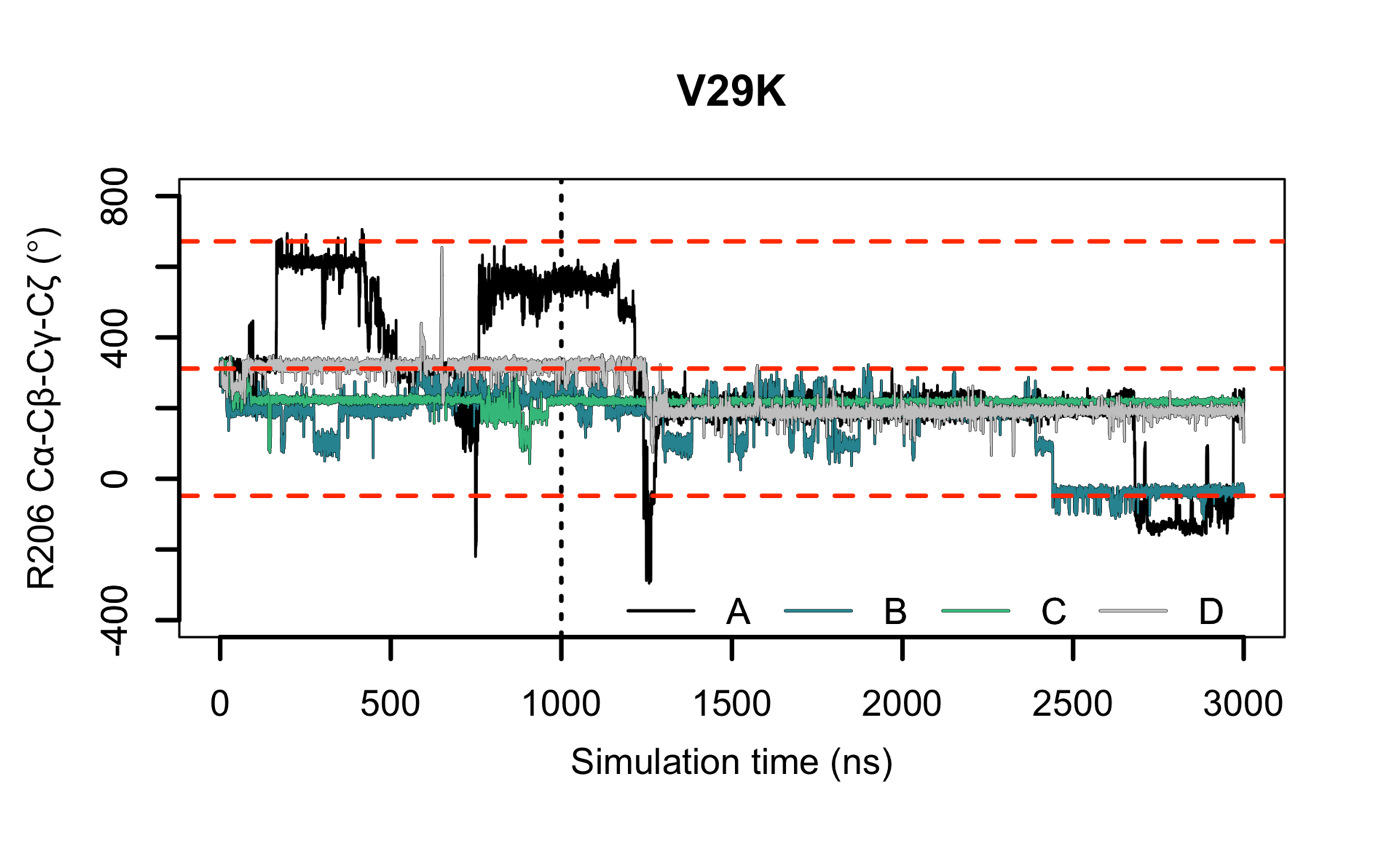


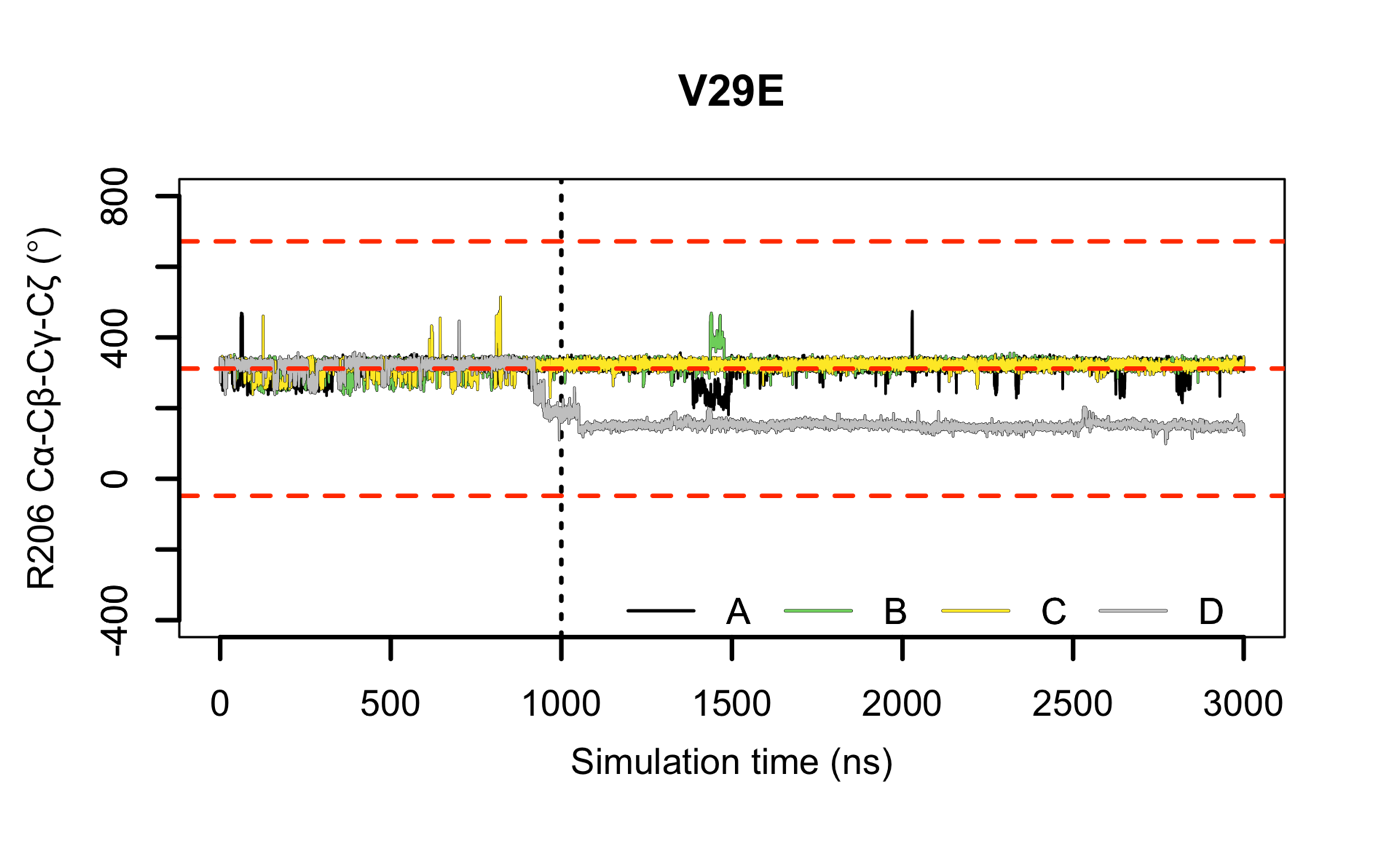


**Figure S12. Time course of the R206^h2.2^ dihedral angle Cα-Cβ-Cγ-Cζ** shown for one set of wt GlpF (top), V29K(middle), and V29E (bottom) simulations. The vertical dotted line separates the equilibration part of the simulation (first µs) and the production part of the simulation (1-3 µs). The red horizontal dashed lines visualize the dihedral angle and its two periodic images in the crystal structure.


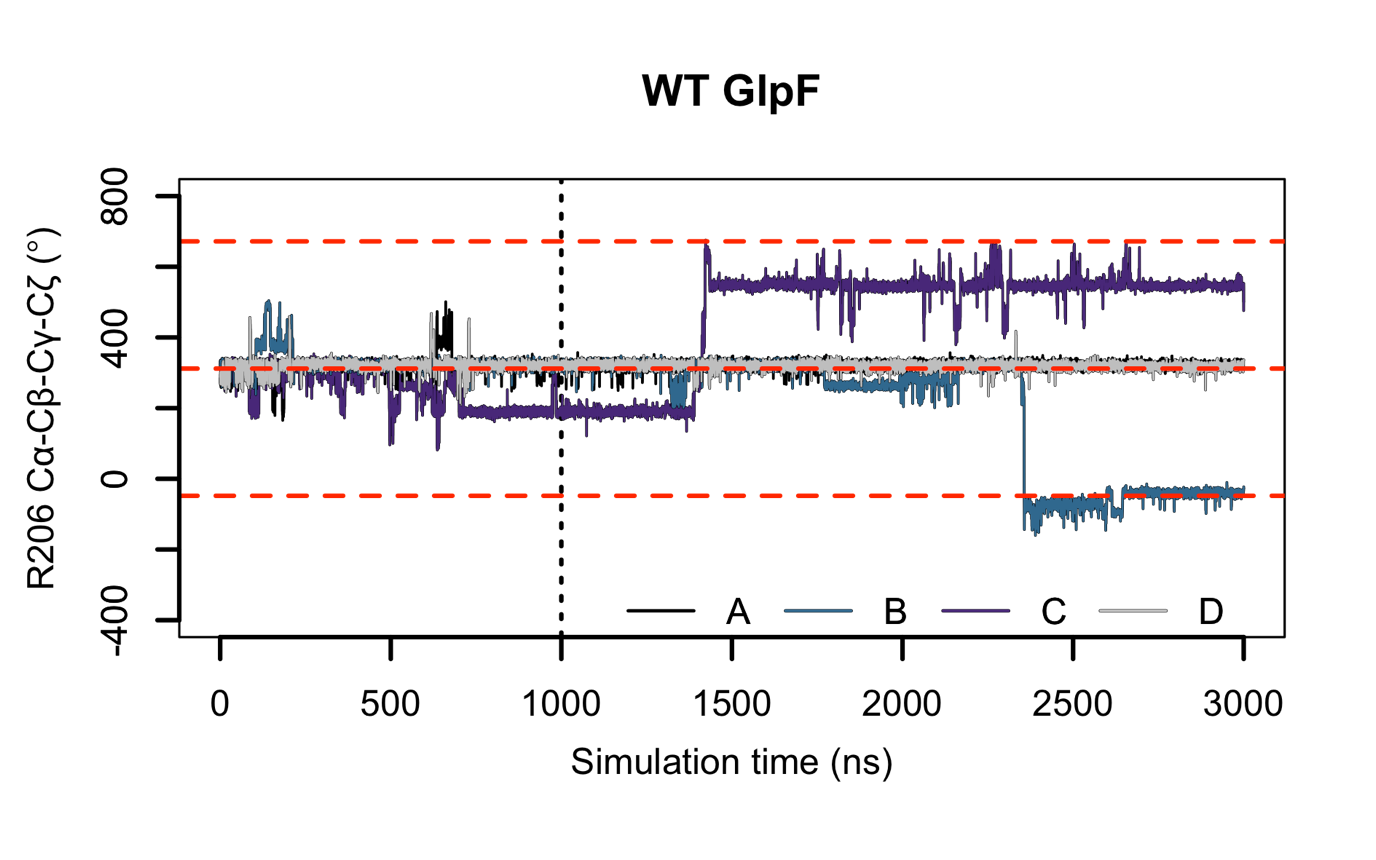

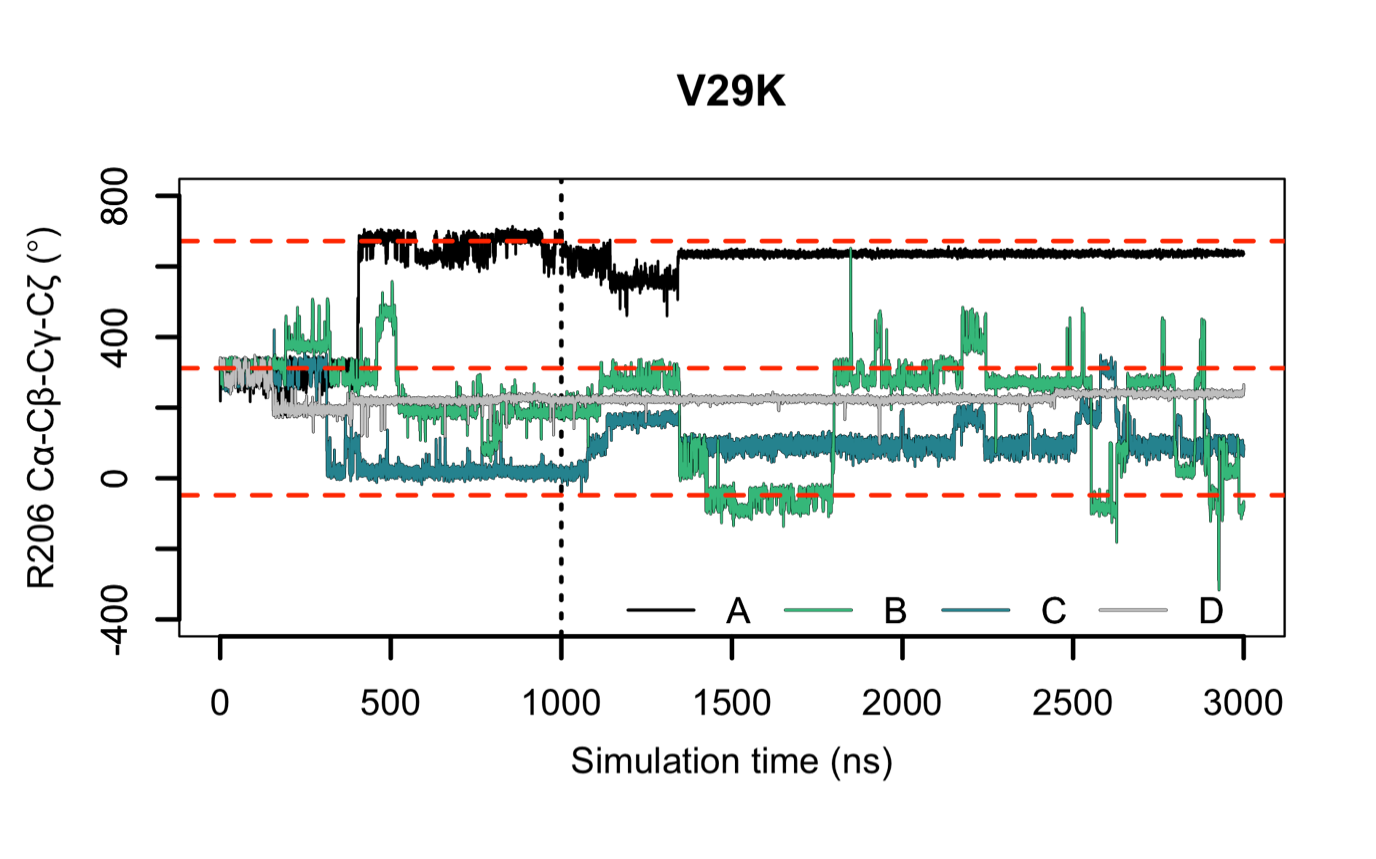

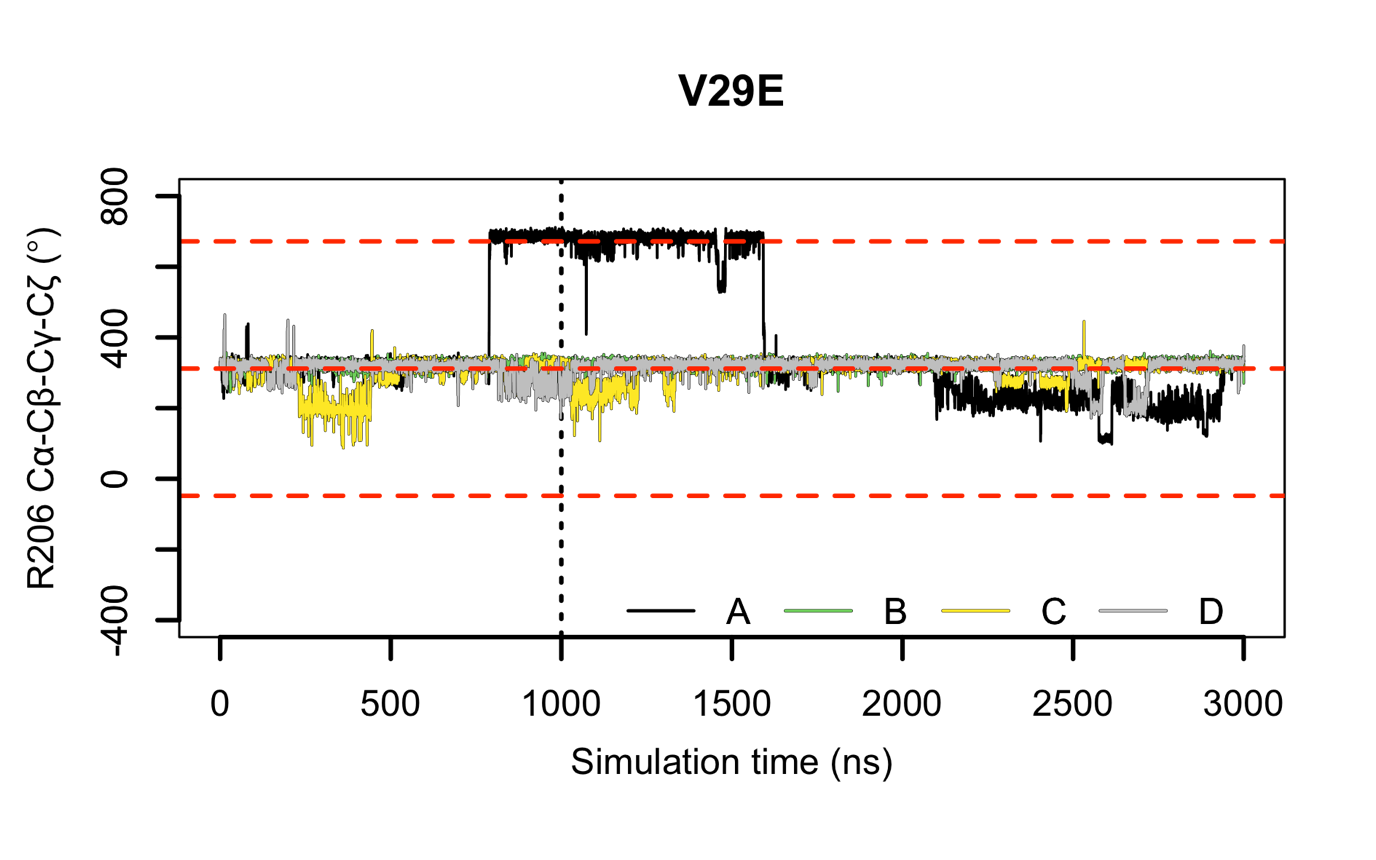


**Figure S13. Time course of the R206^h2.2^ dihedral angle Cα-Cβ-Cγ-Cζ** shown for a second set of wt GlpF (top), V29K(middle), and V29E (bottom) simulations. The vertical dotted line separates the equilibration part of the simulation (first µs) and the production part of the simulation (1-3 µs). The red horizontal dashed lines visualize the dihedral angle and its two periodic images in the crystal structure.


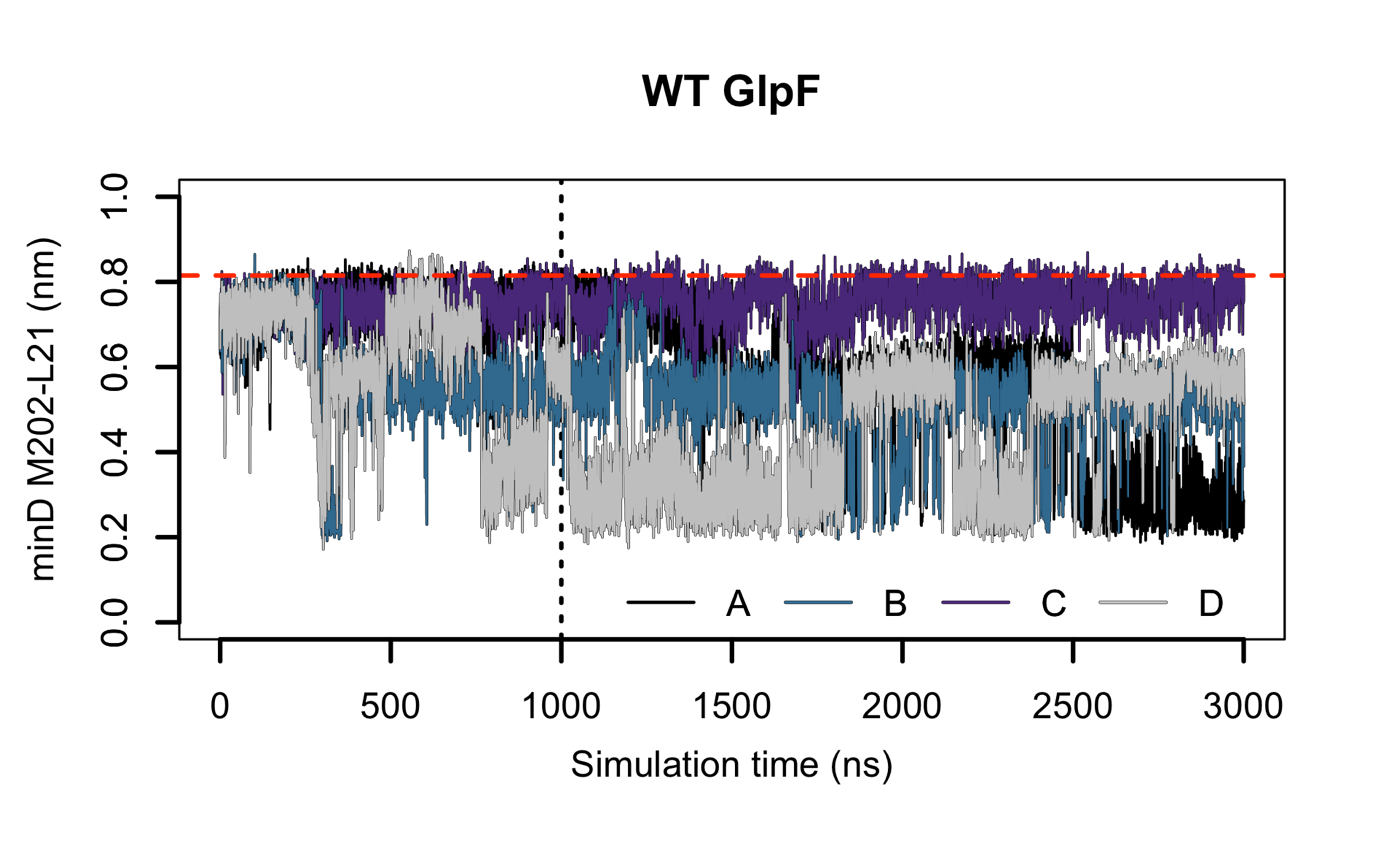

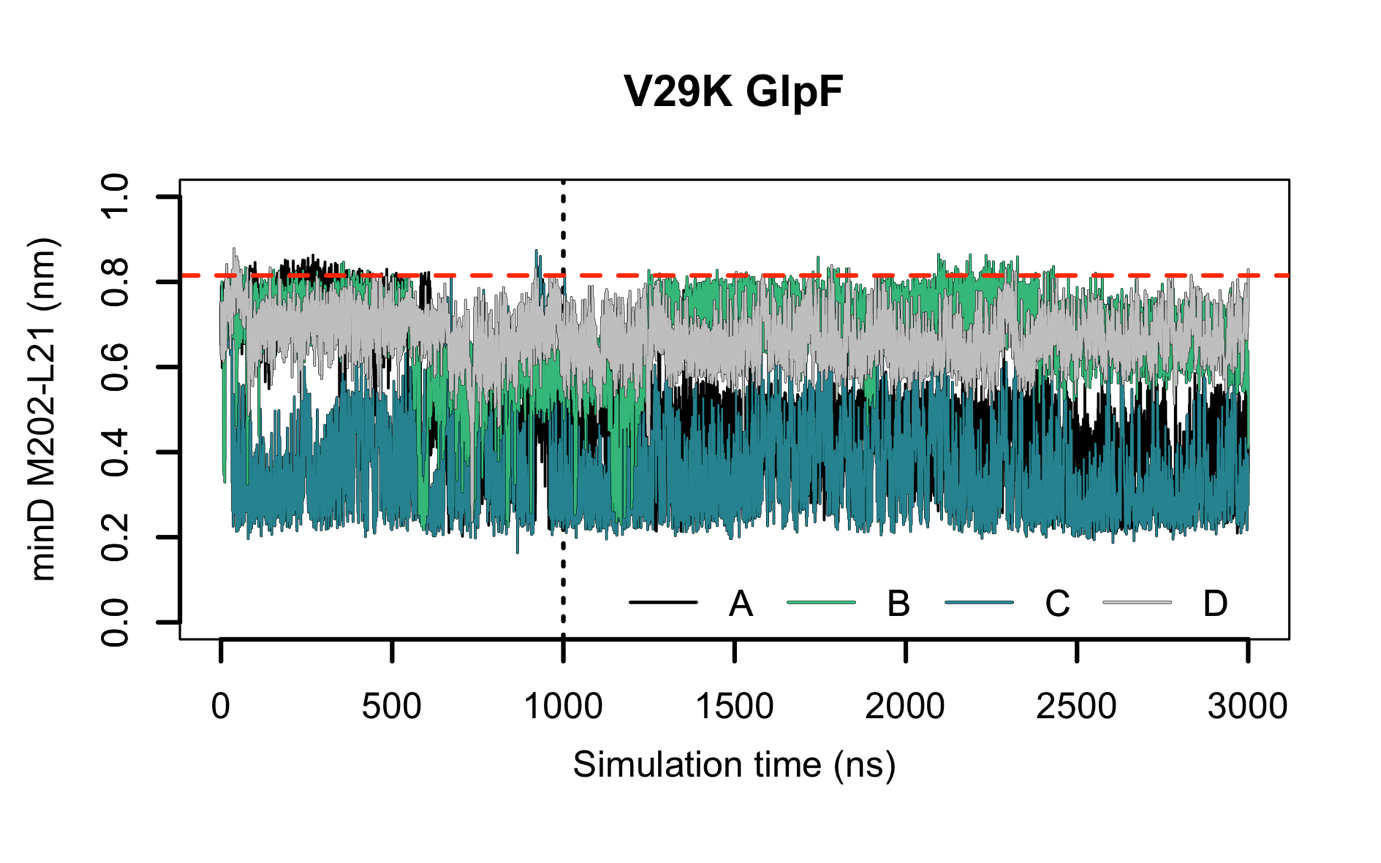

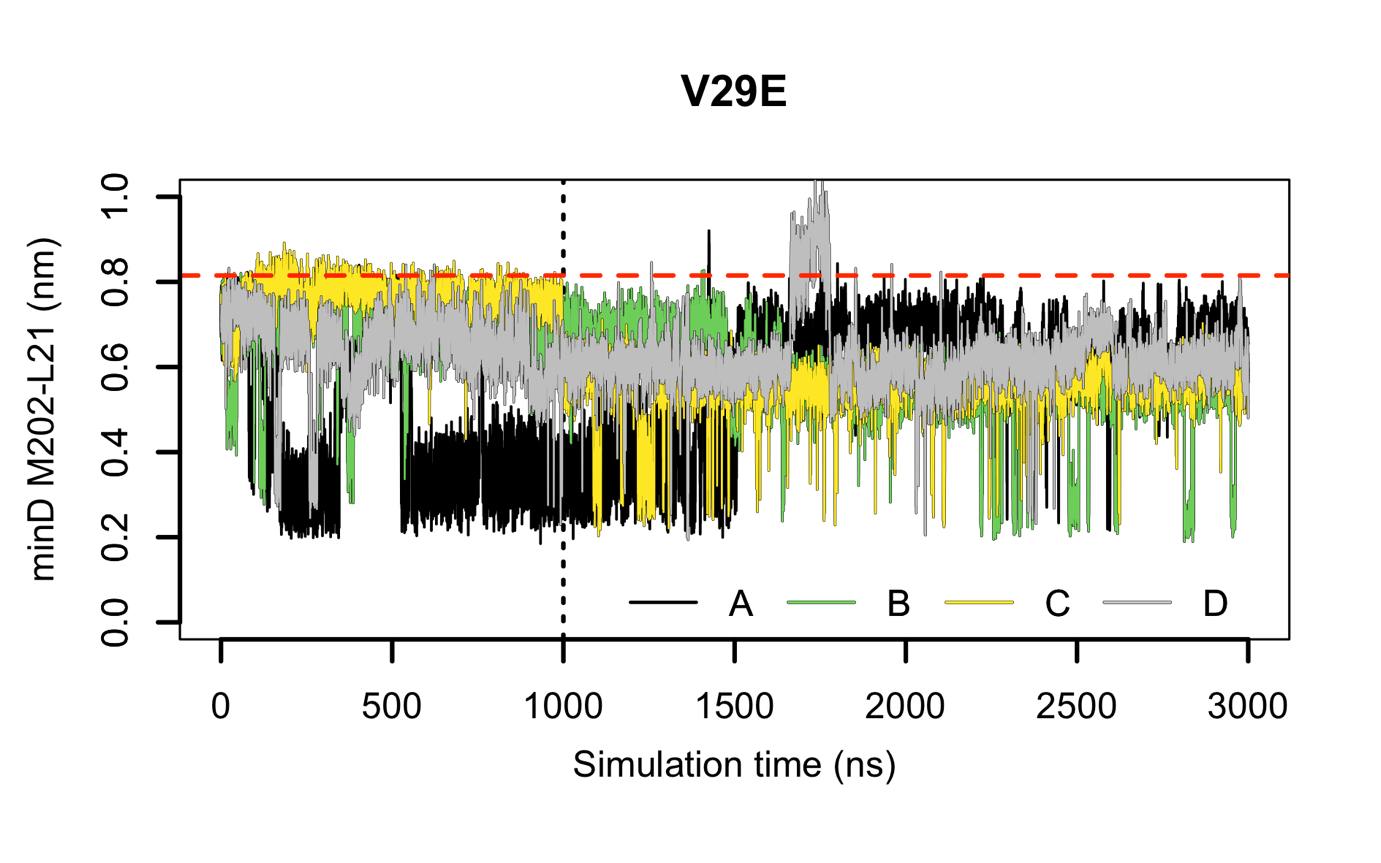


**Figure S14. Time course of the minimal distance between sidechains of M202^h2.-2^ and L21^1.1^** exemplarily shown for wt GlpF (top), V29K (middle), and V29E (bottom), one simulation each. The vertical dotted line separates the equilibration part of the simulation (first µs) and the production part of the simulation (1-3 µs). The red horizontal dashed line visualizes the minimal distance in the crystal structure.


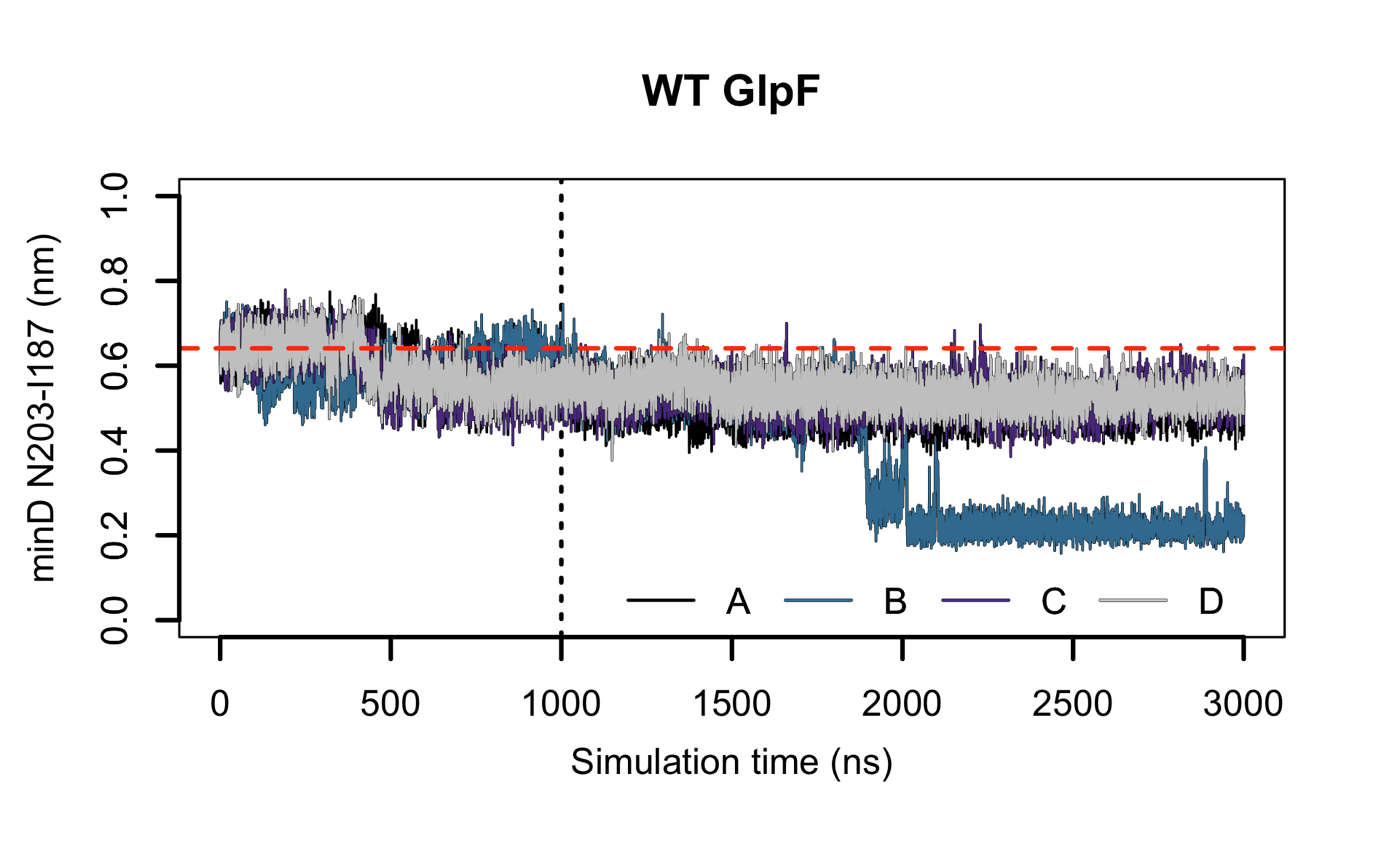

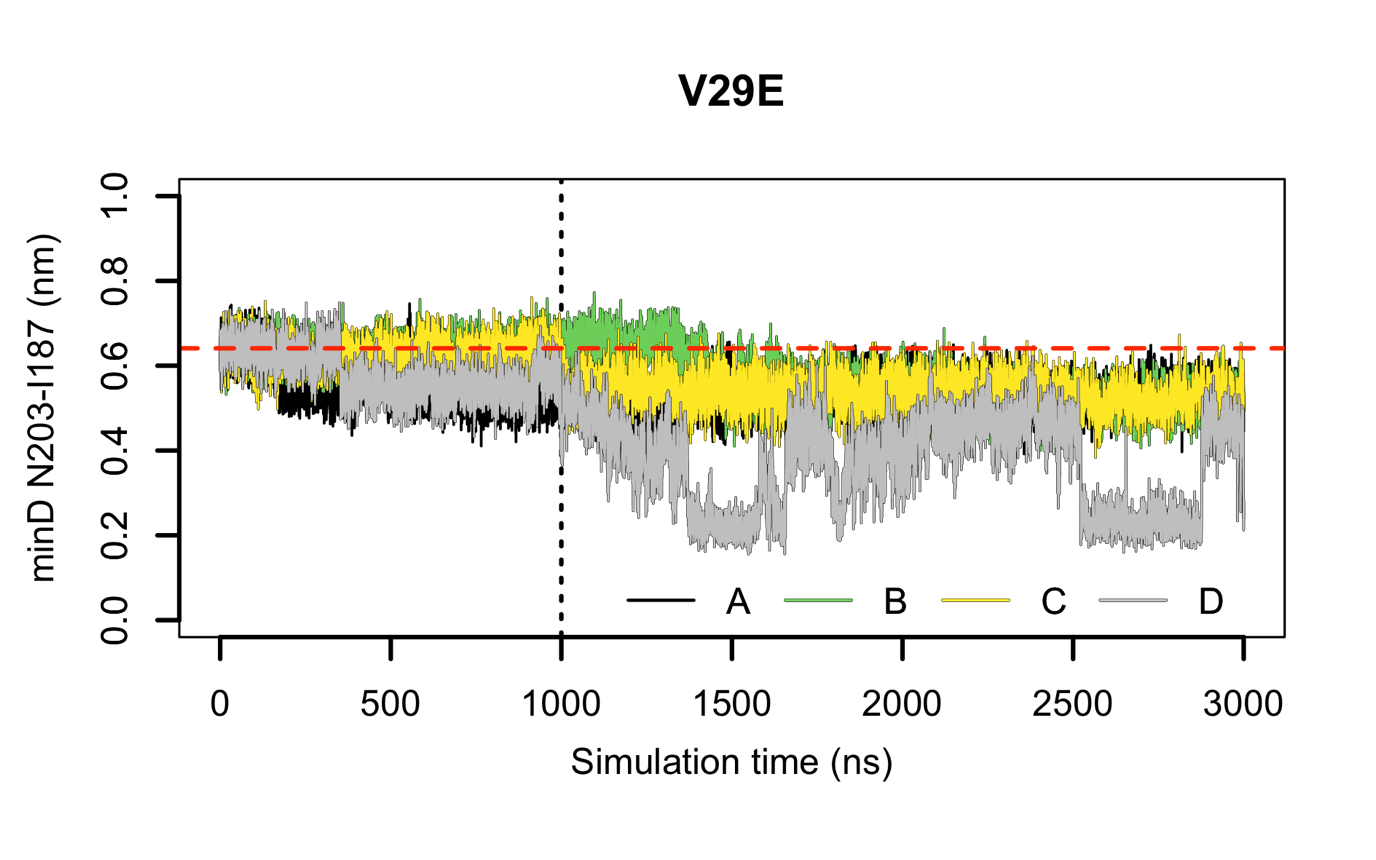


**Figure S15.** **Time course of the minimal distance between sidechains of N203^h2.-1^ and I187^5.1^** exemplarily shown for wt GlpF (top) and V29E (bottom), one simulation each. The vertical dotted line separates the equilibration part of the simulation (first µs) and the production part of the simulation (1-3 µs). The red horizontal dashed line visualizes the minimal distance in the crystal structure.


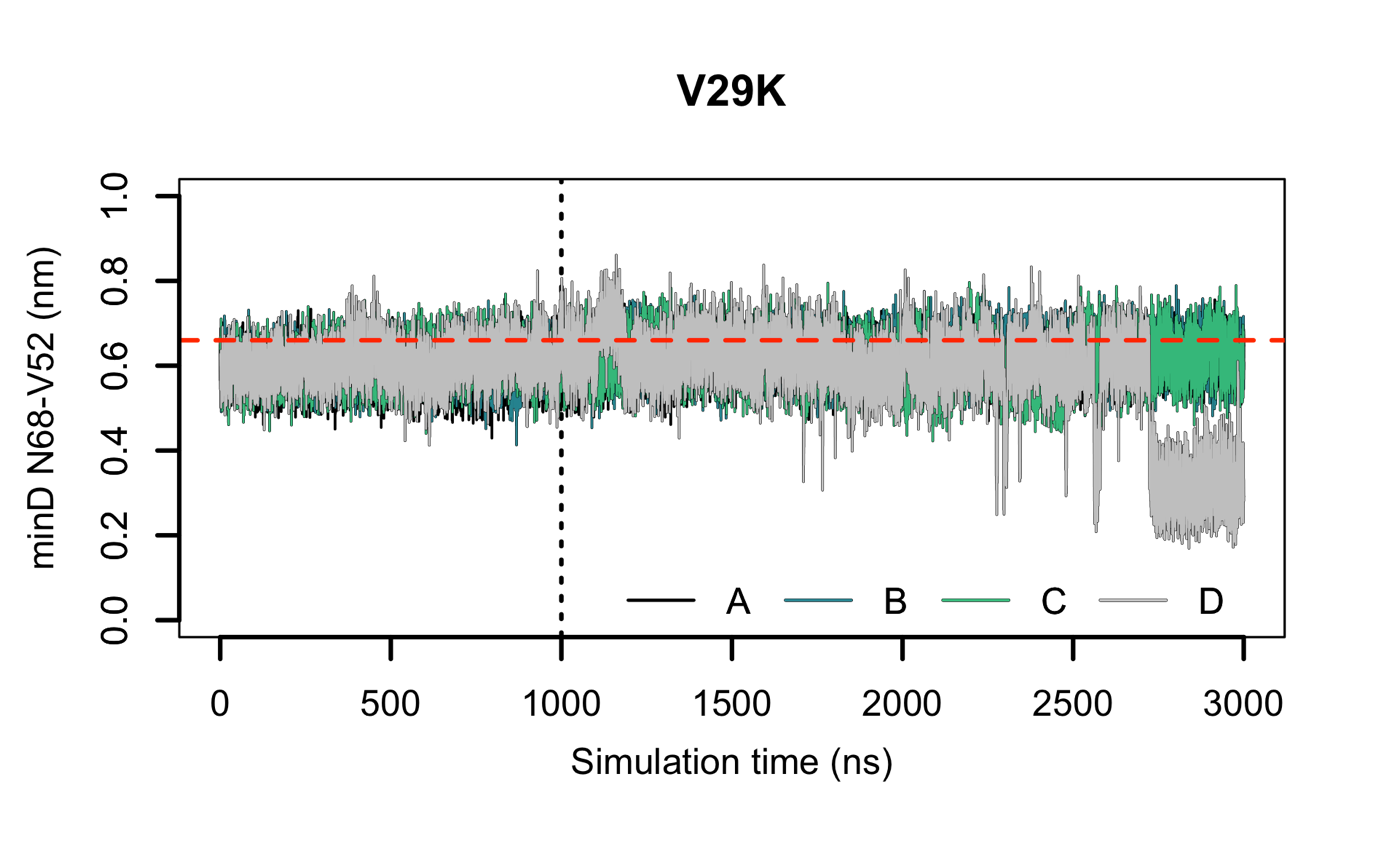

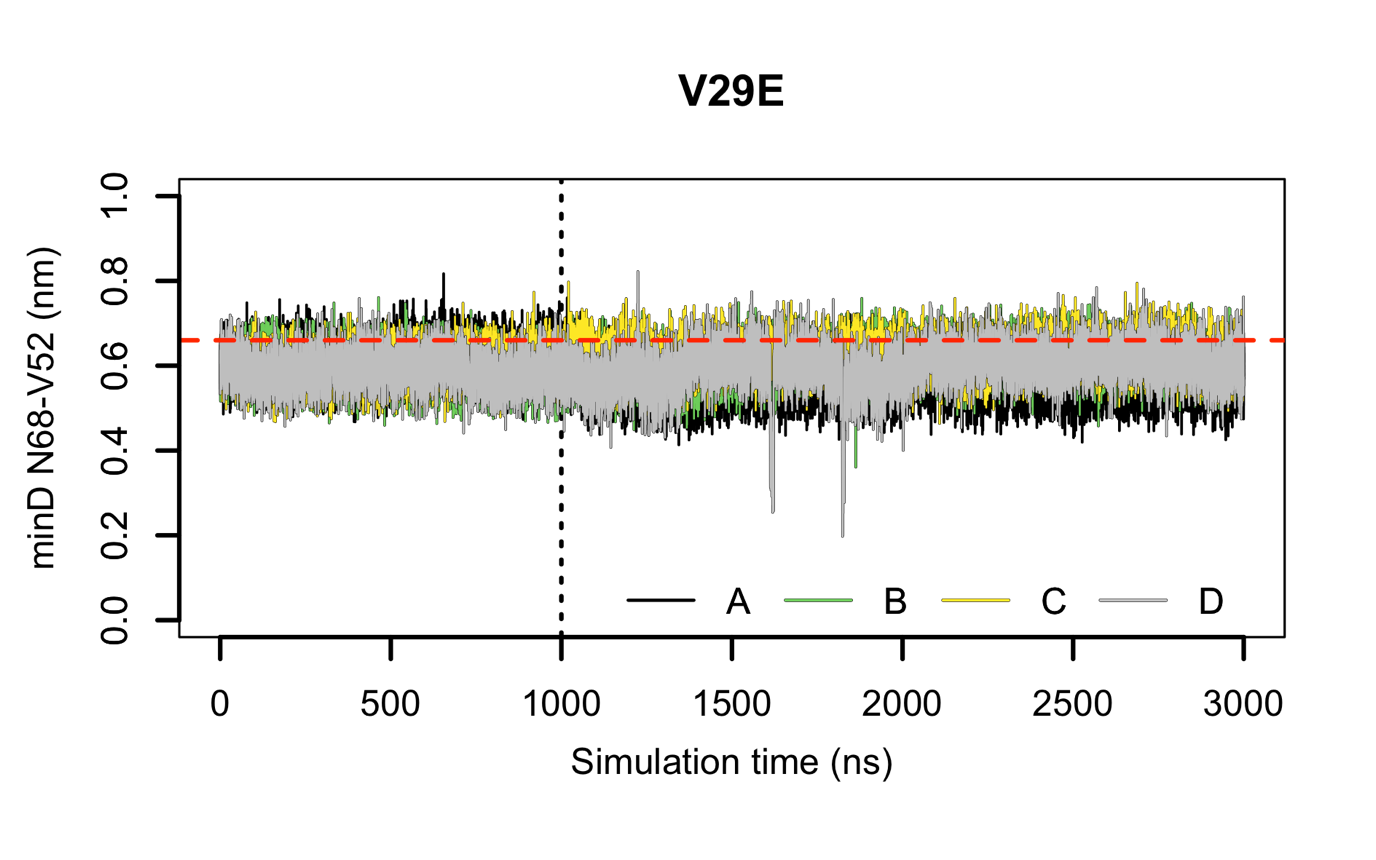


**Figure S16. Time course of the minimal distance between sidechains of N68^h1.-1^ and V52^2.1^** exemplarily shown for V29K (top) and V29E (bottom). The movement can be of persistent nature (V29K chain D), or floppy nature (V29E chain D, V29K chain D). The vertical dotted line separates the equilibration part of the simulation (first µs) and the production part of the simulation (1-3 µs). The red horizontal dashed line visualizes the minimal distance in the crystal structure.

*
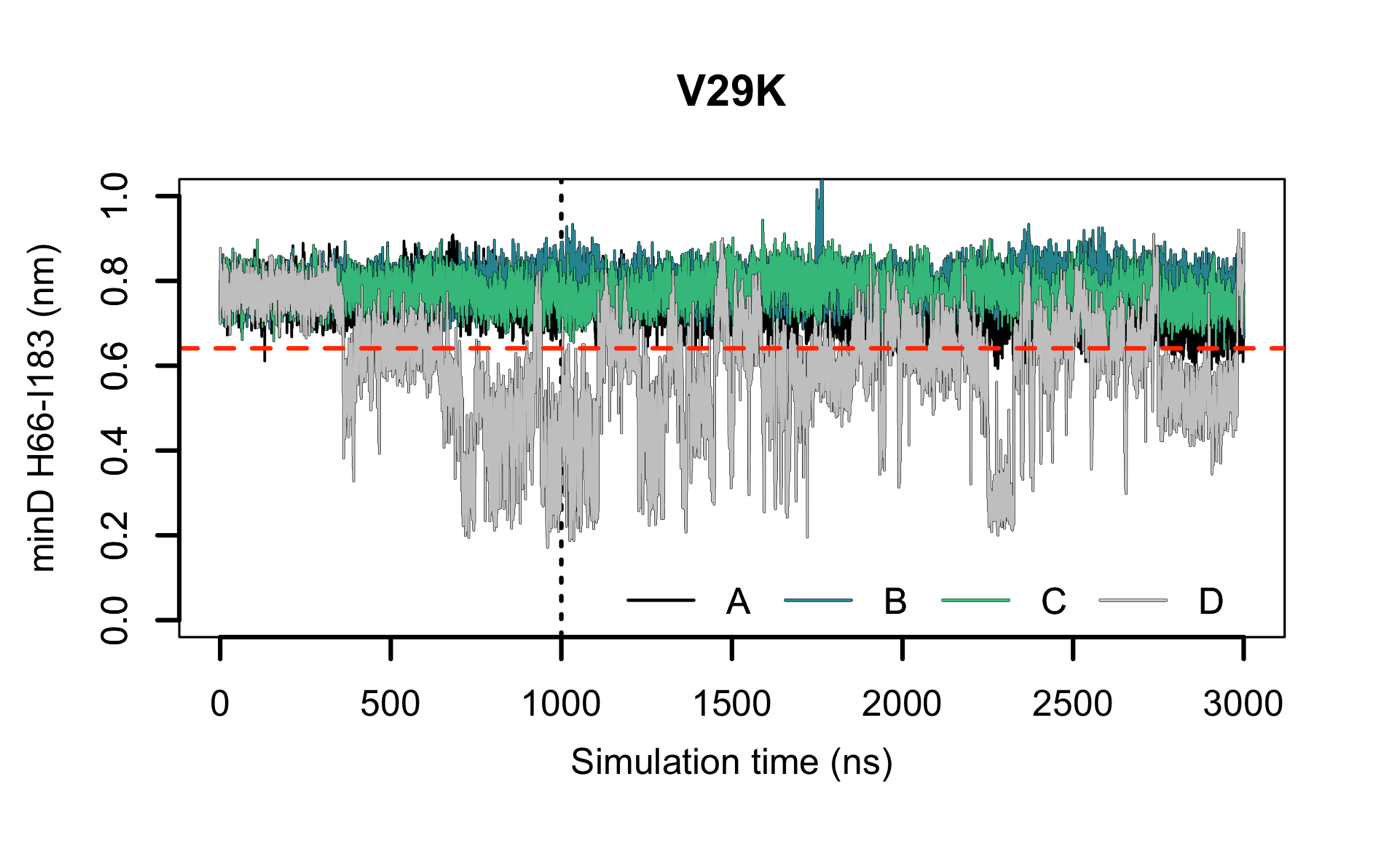
*

**Figure S17. Time course of the minimal distance between sidechains of H66^h1.-3^ and I183^5.-3^** exemplarily shown for V29K. The vertical dotted line separates the equilibration part of the simulation (first µs) and the production part of the simulation (1-3 µs). The red horizontal dashed line visualizes the minimal distance in the crystal structure.


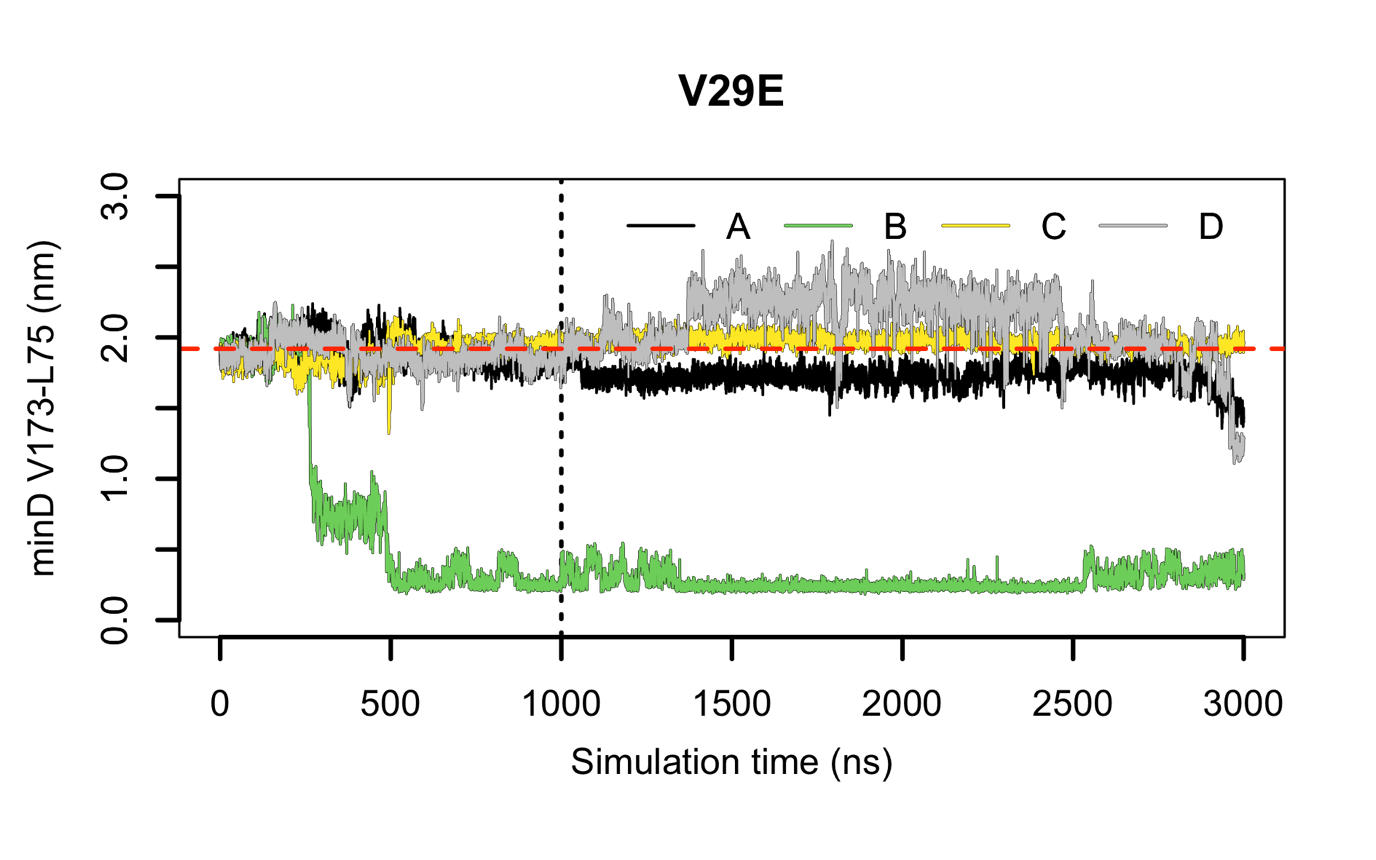


**Figure S18.** **Time course of the minimal distance between sidechains of V173^4.17^ and L75^h1.6^** shown for one V29E simulation, where loop D of chain B moved into the pore lumen. The vertical dotted line separates the equilibration part of the simulation (first µs) and the production part of the simulation (1-3 µs). The red horizontal dashed line visualizes the minimal distance in the crystal structure.

| 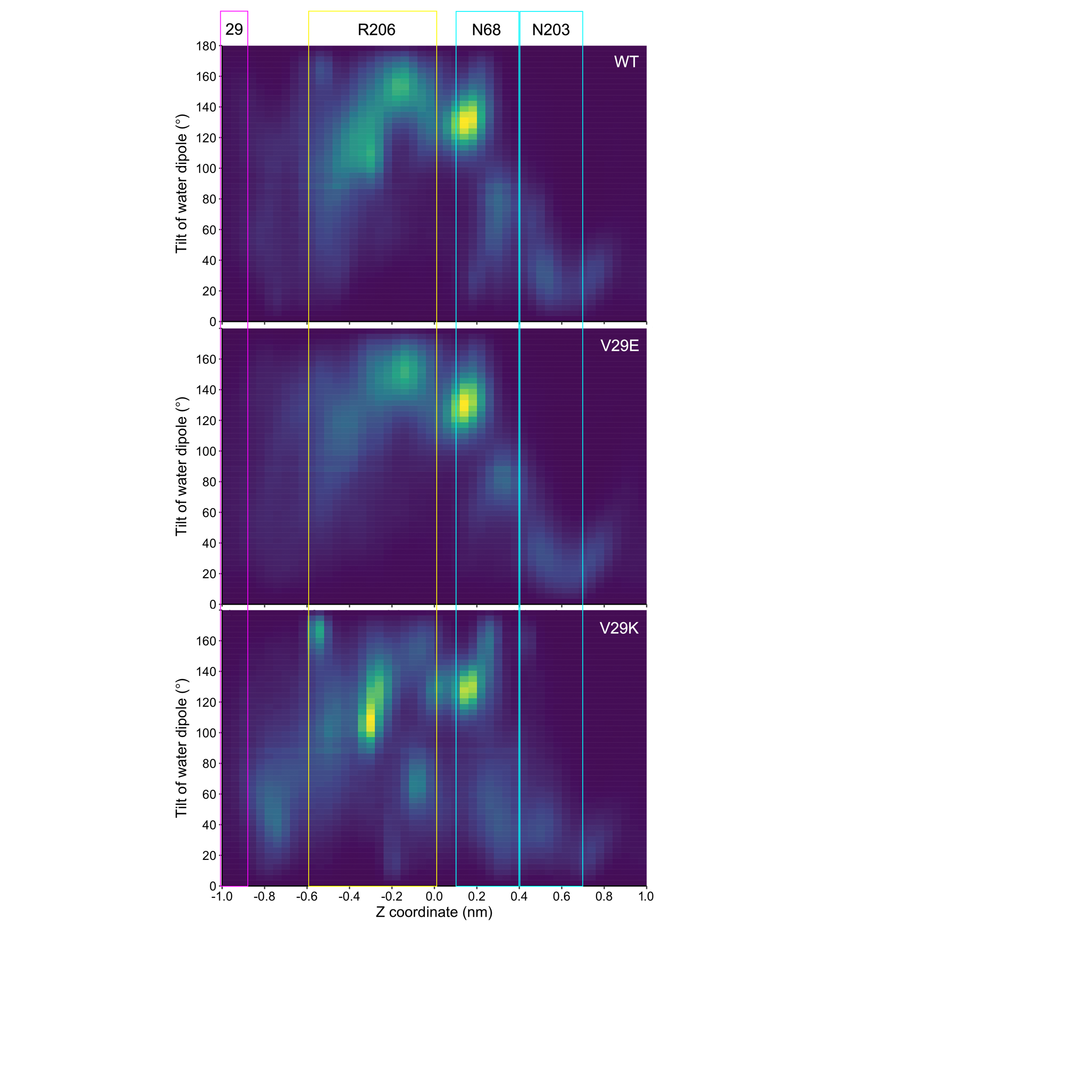 | **Figure S19. Orientation of the dipoles of water molecules passing the pore of wt GlpF and the mutants**. The orientation is described as the tilt of the water dipole to the membrane normal. On the top the localization of important residues (i.e. the mutated residue 29^1.9^, R206^h2.2^ in the ar/R filter and N68^h1.-1^ and N203^h2.-1^ from the NPA filters are indicated. |
| --- | --- |

*
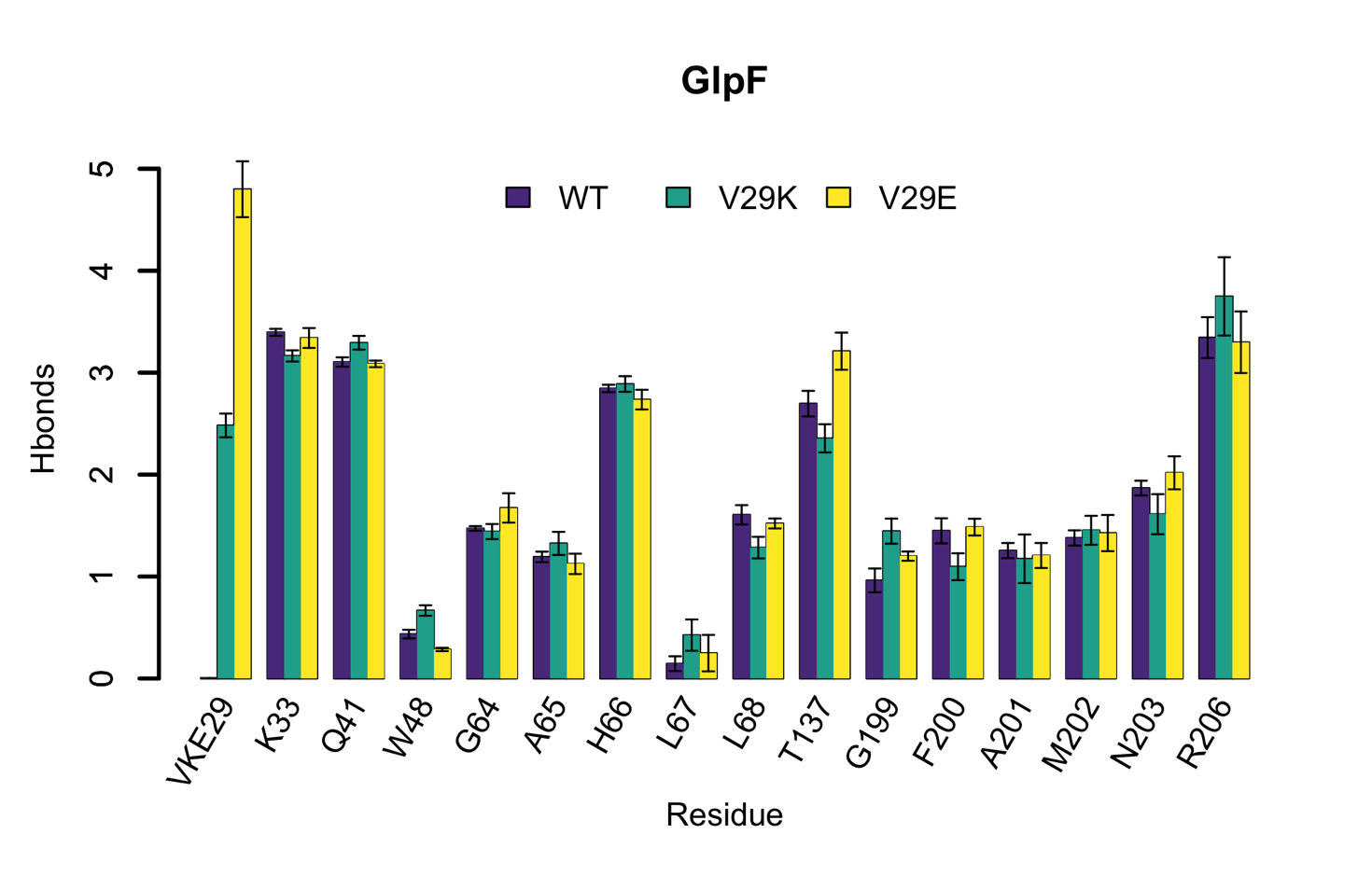
*

**Figure S20. Number of hydrogen bonds between pore lining residues and water.** The residue T137^4.23^ is found in the vicinity of residue 29^1.9^. The barplot shows the average and SEM of the average number of hydrogen bonds (H-bonds) in 1-3µs of the simulation time in each channel and simulations. VKE29 stands for V29 (WT), K29 (V29K mutant) and E29 (V29E mutant).
